# High-salt diet downregulates TREM2 expression and blunts efferocytosis of macrophages after acute ischemic stroke

**DOI:** 10.1186/s12974-021-02144-9

**Published:** 2021-04-12

**Authors:** Mengyan Hu, Yinyao Lin, Xuejiao Men, Shisi Wang, Xiaobo Sun, Qiang Zhu, Danli Lu, Sanxin Liu, Bingjun Zhang, Wei Cai, Zhengqi Lu

**Affiliations:** 1 Department of Neurology, Mental and Neurological Disease Research Center, The Third Affiliated Hospital of Sun Yat-sen University, 600 Tianhe Road, Guangzhou, Guangdong 510630 People’s Republic of China; 2 Center of Clinical Immunology, Mental and Neurological Disease Research Center, The Third Affiliated Hospital of Sun Yat-sen University, 600 Tianhe Road, Guangzhou, Guangdong 510630 People’s Republic of China

**Keywords:** High-salt diet, Stroke, Macrophage, Phagocytosis, Triggering receptor expressed on myeloid cells 2

## Abstract

**Background:**

A high-salt diet (HSD) is one of the major risk factors for acute ischemic stroke (AIS). As a potential mechanism, surplus salt intake primes macrophages towards a proinflammatory phenotype. In this study, whether HSD could blunt the efferocytic capability of macrophages after ischemic stroke, thus exacerbating post-stroke neural inflammation, was investigated.

**Methods:**

Wild-type male C57BL/6 mice were fed with fodder containing 8% sodium chloride for 4 weeks and subjected to transient middle cerebral occlusion (tMCAO). Disease severity, macrophage polarization as well as efferocytic capability were evaluated. Bone marrow-derived macrophages were cultured in vitro, and the impact of high salinity on their efferocytic activity, as well as their expression of phagocytic molecules, were analyzed. The relationships among sodium concentration, macrophage phenotype, and disease severity in AIS patients were explored.

**Results:**

HSD-fed mice displayed increased infarct volume and aggravated neurological deficiency. Mice fed with HSD suffered exacerbated neural inflammation as shown by higher inflammatory mediator expression and immune cell infiltration levels. Infiltrated macrophages within stroke lesions in HSD-fed mice exhibited a shift towards proinflammatory phenotype and impaired efferocytic capability. As assessed with a PCR array, the expression of triggering receptor expressed on myeloid cells 2 (TREM2), a receptor relevant to phagocytosis, was downregulated in high-salt-treated bone marrow-derived macrophages. Enhancement of TREM2 signaling restored the efferocytic capacity and cellular inflammation resolution of macrophages in a high salinity environment in vitro and in vivo. A high concentration of urine sodium in AIS patients was found to be correlated with lower TREM2 expression and detrimental stroke outcomes.

**Conclusions:**

HSD inhibited the efferocytic capacity of macrophages by downregulating TREM2 expression, thus impeding inflammation resolution after ischemic stroke. Enhancing TREM2 signaling in monocytes/macrophages could be a promising therapeutic strategy to enhance efferocytosis and promote post-stroke inflammation resolution.

**Supplementary Information:**

The online version contains supplementary material available at 10.1186/s12974-021-02144-9.

## Background

High-salt intake is positively correlated with blood pressure, blood lipid concentration, circulating alarmins, and other factors leading to detrimental stroke prognosis. The habit of high-salt diet (HSD) has long been considered as a risk factor for acute ischemic stroke (AIS) [1–3]. Therefore, salt restriction is widely accepted as a vital step in efficient lifestyle intervention to prevent new vascular events, especially AIS [4–6]. Nevertheless, in daily clinical practice, we found that it was not always practical for patients to change the long-established high-salt diet. Therefore, there is an unmet need to develop therapeutic strategies to tackle the already existing high salinity and the associated pathophysiology.

Macrophage, as an innate immune cell, has a high degree of parallelism and flexibility [7, 8]. Macrophages could release multiple proinflammatory mediators or efficiently sweep cell debris and promote neural recovery. The phenotypic shift of macrophages depends largely on the cues of the microenvironment [9, 10]. Recent research has elucidated that surplus dietary salt shifts macrophages/microglia towards the classical activated proinflammatory phenotype, which is often referred to as M1 [11], indicating that excessive salt intake breaks the M1/M2 macrophages balance and further aggravates the inflammatory response. In vivo, the proinflammatory property of macrophages in HSD-fed mice contributed to blood-brain barrier (BBB) disruption after stroke and exacerbated stroke outcomes [12]. Efferocytosis represents a vital anti-inflammatory process of macrophages. Timely clearance of cell debris and cell corpses is essential for subsequent tissue reconstruction of lesions [9, 13, 14]. Nevertheless, the impact of HSD on phagocytic activity and the subsequent anti-inflammatory functions of macrophages remain elusive.

The current study investigated the impact of excessive salt intake on the efferocytic capacity of macrophages after ischemic stroke. Our data indicated that HSD downregulated the expression of the phagocytic molecule triggering receptor expressed on myeloid cells 2 (TREM2) in macrophages, thus impeding their debris clearing activities. Enhancement of TREM2 signaling in macrophages rescued the inflammation resolving functions and displayed delightful therapeutic effects. TREM2 could be a promising therapeutic target of AIS, especially in patients with un-converted HSD habits.

## Methods

### Ethical statement

The clinical and experimental animal studies were approved by the Medical Ethics Committee of the Third Affiliated Hospital of Sun Yat-Sen University and the Animal Care and Use Committee of Sun Yat-Sen University, respectively. All participants had signed the informed consent according to the principles illustrated in the Declaration of Helsinki.

### Patients

In this study, a total of 38 stroke patients recruited in The Third Affiliated Hospital of Sun Yat-Sen University from July 2018 to October 2019 consecutively had an independently documented primary stroke event in combination with confirmed magnetic resonance imaging (MRI) evidence showing ischemic stroke. All patients recruited in the study met the following inclusion criteria: (i) onset age ≥ 18 years; (ii) first symptomatic ischemic stroke; (iii) clinical evidence of motor, language, attention, visual, or memory deficits based on neurological examination; (iv) time of enrolment: < 7 days from stroke onset. Exclusion criteria included (i) prior stroke, (ii) cerebral hemorrhage, (iii) malignant tumors, (iv) patent foramen ovale, (v) vasculitis, (vi) autoimmune disease, (vii) chronic kidney disease with hemodialysis, (viii) Parkinson’s disease, (ix) treatment with systemic glucocorticoids or other immunosuppressive agents within 14 days of admission, and (x) thromboembolism, collagen disease, disseminated intravascular coagulation, and advanced liver disease [15, 16]. Clinical data, including age, gender, and scores of the National Institute of Health Stroke Scale (NIHSS), were recorded. We estimated dietary sodium intake by measurements of a 24-h urinary excretion of sodium. Patients were instructed to collect their 24-h urine void on the day of their clinic visit, and the 24-h sodium excretion was calculated by multiplying the sodium concentration in the urine by the total urine volume during this period. Patient demographics, including comorbidities, were summarized in Supplementary Table 1.

### MRI scanning and infarct volume analysis of patients

Magnetic resonance imaging (MRI) was performed within 24h of admission using 1.5- or 3.0-T magnetic resonance imaging (Sigma; GE Medical Systems, Milwaukee, WI, USA). In this study, the diffusion-weighted imaging (DWI) spin-echo planar sequence included 20 contiguous axial oblique slices (*b* = 0 and 1000 s/mm^2^ isotropically weighted; repetition time/echo time, 6000/60.4 ms; acquisition matrix, 128 × 128; slice thickness, 5mm; interstice gap, 1 mm; field of view, 24 cm). DWI lesions in 38 patients were measured with Analyze 7.0 software (Analyze Direct, KS). Cerebral infarct size was assessed based on the largest infarct diameter determined on the image demonstrating the largest lesion [17–19]. MRI scans of patients were assessed by the experienced neurologist Zhengqi Lu, who was blinded to the patients’ clinical features. All images were interpreted with the same window settings, monitor types, and lighting conditions.

### PBMC isolation

Anti-coagulated blood (3 mL) was collected and then diluted 2-fold with PBS, pipetted into a centrifuge tube prefilled with Ficoll lymphocyte separation solution (TBDscience), followed by centrifuged at 2000 rpm for 25 min at room temperature without deceleration. PBMCs from the buffy coat were washed twice with PBS and then stored at − 80°C until further analysis.

### Animals

C57BL/6 wild-type mice (8 weeks old, weight 18–25 g) were purchased from the Guangdong Medical Laboratory Animal Center (Guangzhou, China) and housed in a humidity- and temperature-controlled animal facility at Sun Yat-sen University with a 12-h light-dark cycle. Mice received normal chow (0.5% NaCl) and tap water ad libitum (normal diet) or sodium-rich chow (8% NaCl) and tap water containing 1% NaCl ad libitum (HSD) for 4 weeks, according to the experiment.

### Model of acute ischemic stroke

Mice were subjected to focal acute ischemic stroke induced with transient middle cerebral artery occlusion (tMCAO). Procedures for tMCAO were described previously [15]. Briefly, mice were anesthetized with 1.5–2.0% isoflurane under conditions of spontaneous breathing. A filament was inserted into the external carotid artery (ECA) and was directed to the middle cerebral artery (MCA) through the internal carotid artery (ICA). Filament insertion into the ICA was maintained for 60 min followed by reperfusion with the maintenance of core body temperatures. Cerebral blood flow (CBF) during surgery was measured by laser Doppler flow cytometry. Mice with a more than 70% reduction of blood flow in the ischemic core were included in the study, and mice that died during surgery were excluded. Survival of mice was recorded.

### Infarct volume analysis

For immunologic staining of NeuN, six equally spaced coronal brain sections encompassing the MCA territory were stained with NeuN antibodies. Infarct volume in NeuN-stained sections was analyzed with NIH ImageJ software. The infarct area was determined as the difference between the NeuN-positive area of contralateral hemispheres and ipsilateral hemispheres. Brain infarct was determined by multiplying the mean area of tissue loss by the distances between two adjacent stained brain slices.

### Primary macrophage enriched culture and stimulation

Primary macrophage-enriched cultures were prepared from the bone marrow of 6- to 8-week-old healthy C57BL/6 wild-type mice using the EasySep Mouse Monocyte Enrichment Kit (Stem Cell) according to manufacturer’s instructions. Macrophages were induced with MCSF (50 ng/ml) for 6 days in macrophage culture medium (RPMI1640 + 10%FBS). For polarization, macrophages were treated with lipopolysaccharide (LPS, 100 ng/mL, Sigma) or IL-4 (20 ng/ml, Peprotech) for 24 h.

### Macrophage depletion and transfer

A single dose of clodronate liposomes (Liposoma, 10 ml/kg, i.p.) was administered to mice at 3 days before tMCAO to deplete peripheral monocytes/macrophages. Empty liposomes were used as control (Liposoma, 10 ml/kg, i.p.). Depletion efficacy of clodronate liposomes was confirmed with flow cytometric analysis. Bone marrow-derived macrophages (BMDM, 2 × 10^6^) were transferred to the tMCAO model intravenously immediately after reperfusion.

### Primary microglia culture

Primary mouse microglia were obtained from BLUEFBIO company and cultured in culture medium (DMEM-HG + 10%FBS) until treatment.

### Primary cortical neuron culture and oxygen-glucose deprivation

Primary cortical neuronal cultures were prepared from E16-18 embryos of C57BL/6 mice as previously described [20].

Neuronal ischemia was induced with oxygen-glucose deprivation (OGD). Briefly, the culture medium (neural basal medium + B27 + 2% glutamate) was removed and replaced by EBSS (Gibco). Neurons were then incubated in 95% N_2_ + 5% CO_2_ for 90 min.

### Phagocytosis assay

For evaluation of efferocytic capacity, apoptotic neurons were labeled with the dead cell marker propidium iodide (PI) in PBS (1 μg/ml, 37 °C, 15 min) and treated with macrophages at a ratio of dead neurons:macrophages = 5:1, for the indicated time periods. For in vitro immunostaining experiments, macrophages were pre-grown on poly-l-lysine-coated coverslips. The coverslips of macrophages were washed two times to remove unengulfed neurons and fixed with 4% paraformaldehyde. The coverslips were then subjected to immunostaining and removed from wells using tweezers and mounted to the slides. F-actin in macrophage was then stained with Alexa Fluor488 phalloidin (A12379, 1:500 in PBS; Invitrogen) at room temperature in the dark for 30 min. For the flow cytometry experiment, macrophages were pre-seeded on 24-well plates and treated with the same ratio of dead neurons for indicated time points. Macrophages were washed with PBS, detached from wells with trypsin, and subjected to flow cytometric analysis. The percentage of efferocytic macrophages (PI^+^) was calculated via flow cytometric analysis.

### Lentiviral infection of macrophage

Lenti virus was constructed and packaged by FenghBio (Changsha, China). The cultured macrophages were infected for 3 days with Lenti-TREM2 or the control vectors. Overexpression of TREM2 was confirmed by western blot and flow cytometry.

### Flow cytometric analysis

The brain tissue was homogenized and prepared as single-cell suspensions for flow cytometric analysis (FACS). Briefly, brains were dissected, and ipsilateral hemispheres were collected. Each hemisphere was subjected to digestion with 0.25% trypsin-EDTA (Thermo Fisher, Carlsbad, CA, USA) at 37 °C for 25 min. The brain tissue was then pressed through a cell strainer (70 μm). Brain cells were separated from myelin debris by centrifugation in 30%/70% Percoll solution (GE Healthcare Biosciences AB, Uppsala, Sweden). Brain cells at the interface were collected, washed with HBSS, and subjected to further staining. The following antibodies were used: CD45-PE-Texas Red (1:400, BioLegend), CD11b-PE (1:400, BioLegend), CD3-PerCp/Cy5.5 (1:400, BioLegend), CD19-FITC (1:400, BioLegend), Ly6G-APC/Cy7 (1:400, BioLegend), TREM2-PE (1:200, R&D Systems), TNFα-PE (1:200, BioLegend), CD206-Alexa Fluor 647 (1:200, BD bioscience), and Arg1-APC (1:200, R&D Systems). FACS was performed using a fluorescence-activated cell sorter flow cytometer (BD bioscience, San Diego, CA), and data were analyzed using FlowJo X 10.0.7r2 software. Appropriate isotype controls were stained following the manufacturer’s instructions (Thermo Fisher, Carlsbad, CA, USA). Fluorochrome compensation was performed with single-stained OneComp eBeads (Thermo Fisher, Carlsbad, CA, USA). For data presentation, when cells could be divided into negative or positive populations, the percentage of cells was calculated. When the expression of the coordinated marker was consecutive and population separation was obscure, data were presented as mean fluorescence intensity (MFI).

### Immunofluorescence staining and cell quantification

Animals were euthanized and perfused with PBS followed by 4% paraformaldehyde. After sufficient perfusion, the brains were removed and then cut into 25-μm frozen cryo-sections using a microtome. Brain sections were incubated with primary antibodies at 4°C overnight. After being washed with PBS, sections were incubated with secondary antibodies for 1h at room temperature. Sections were then washed and mounted with DAPI Fluoromount-G (Thermo Fisher, Carlsbad, CA, USA). The following primary antibodies were used: rabbit anti-NeuN (1:500, Abcam), rabbit anti-Iba1 (1:1000, Wako Pure Chemical Industries), goat anti-Iba1 (1:500, Abcam), rabbit anti-SIK1 (1:500, Proteintech), goat anti-CD206 (1:500, R&D Systems), and rat anti-CD16 (1:500, Santa Cruz Biotechnology) antibodies. The following secondary antibodies were applied: anti-rabbit secondary antibody conjugated with Cy3 (1:1000, Jackson ImmunoResearch Laboratories), anti-rabbit secondary antibody conjugated with Alexa Fluor 488 (1:1000, Jackson ImmunoResearch Laboratories), anti-rabbit secondary antibody conjugated with Alexa Fluor 405 (1:1000, Jackson ImmunoResearch Laboratories), anti-goat secondary antibody conjugated with Cy3 (1:1000, Jackson ImmunoResearch Laboratories), anti-goat secondary antibody conjugated with Alexa Fluor 488 (1:1000, Jackson ImmunoResearch Laboratories), and anti-rat secondary antibody conjugated with Alexa Fluor 488 (1:1000, Jackson ImmunoResearch Laboratories). For neuronal apoptosis analysis, terminal deoxynucleotidyl transferase dUTP nick end labeling (TUNEL) was processed after NeuN labeling according to the manufacturer’s instructions (Thermo Fisher). Confocal microscopy images were acquired using a Leica SP confocal microscope and Leica confocal software. Immunopositive cell quantification and area analysis were performed with the NIH ImageJ software by an investigator who was blinded to the experimental design. In quantification of cells in the stroke penumbra, the stroke core was identified as the region in which the majority of DAPI-stained nuclei were shrunken, and the stroke penumbra was defined as the region of generally morphologically normal cells, approximately 450–500 μm wide, surrounding the stroke core.

### Quantitative determination of mRNA expression

Total RNA from cells was extracted with a commercial kit (ESscience) according to the manufacturer’s instructions. A total of 1ug RNA (OD 260 nm/280 nm = 1.8–2.2) was utilized for the first-strand cDNA synthesis in a 40-μl system using a PrimeScript RT reagent kit (Takara). Real-time polymerase chain reaction (RT-PCR) was performed on a QuantStudio 5 (ABI) quantitative PCR machine using a TB green Premix Ex Taq kit (Takara) with 1 μl of the synthesized cDNA in each reaction with the addition of ROX. The following program was performed: 95 °C for 30 s; 95 °C for 5 s and 60 °C for 34 s, repeated for 40 cycles; 95 °C for 15 s, 60 °C for 1 min and 95 °C for 15 s (Melt curve). Primers used in the study are listed in Supplementary Table 2. Double delta CT was calculated, and the data were presented as fold change normalized to PBS-treated contralateral brain, PBS-treated macrophage, or negative control lentivirus-treated macrophage. Glyceraldehyde-3-phosphate dehydrogenase (GAPDH) was used as a housekeeping gene for normalization. In the data analysis in Fig. 2, Fig. 5, and Supplementary Figure 1D, the mRNA expression level was visualized with the heat map and clustered with the software of R using the “pheatmap” package.

### Western blot

Protein isolation was performed as previously described [21]. Western blots were performed using the standard SDS-polyacrylamide gel electrophoresis method and enhanced chemiluminescence detection reagents (GE Healthcare Biosciences AB, Uppsala, Sweden). Antibodies against TREM1 (1:1000, Abcam), TREM2 (1:1000, Abcam), TNFα (1:1000, Proteintech), IL-10 (1:1000, Proteintech), β-actin (1:3000, Abcam), SIK1 (1:1000, Proteintech), and GAPDH (1:3000, Cell Signaling Technology) were used according to the manufacturer’s directions. Immunoreactivity was semi-quantitatively measured via densitometric gel scanning and analyzed using the MCID image analysis system (Imaging Research, Inc.).

### Statistical analysis

All results were presented as mean ± standard error of the mean (*SEM*). Differences in the means among multiple groups were analyzed using one- or two-way analysis of variance (*ANOVA*). When *ANOVA* showed significant differences, pair-wise comparisons between means were tested by *Dunnett’s test*. The *Student’s t test* was used for comparisons between two groups. The software used for statistical analysis was R v3.6.3. In all analyses, *P* < 0.05 was considered statistically significant.

## Results

### Excess salt intake exacerbates disease outcomes of ischemic stroke

**Fig. 1:**
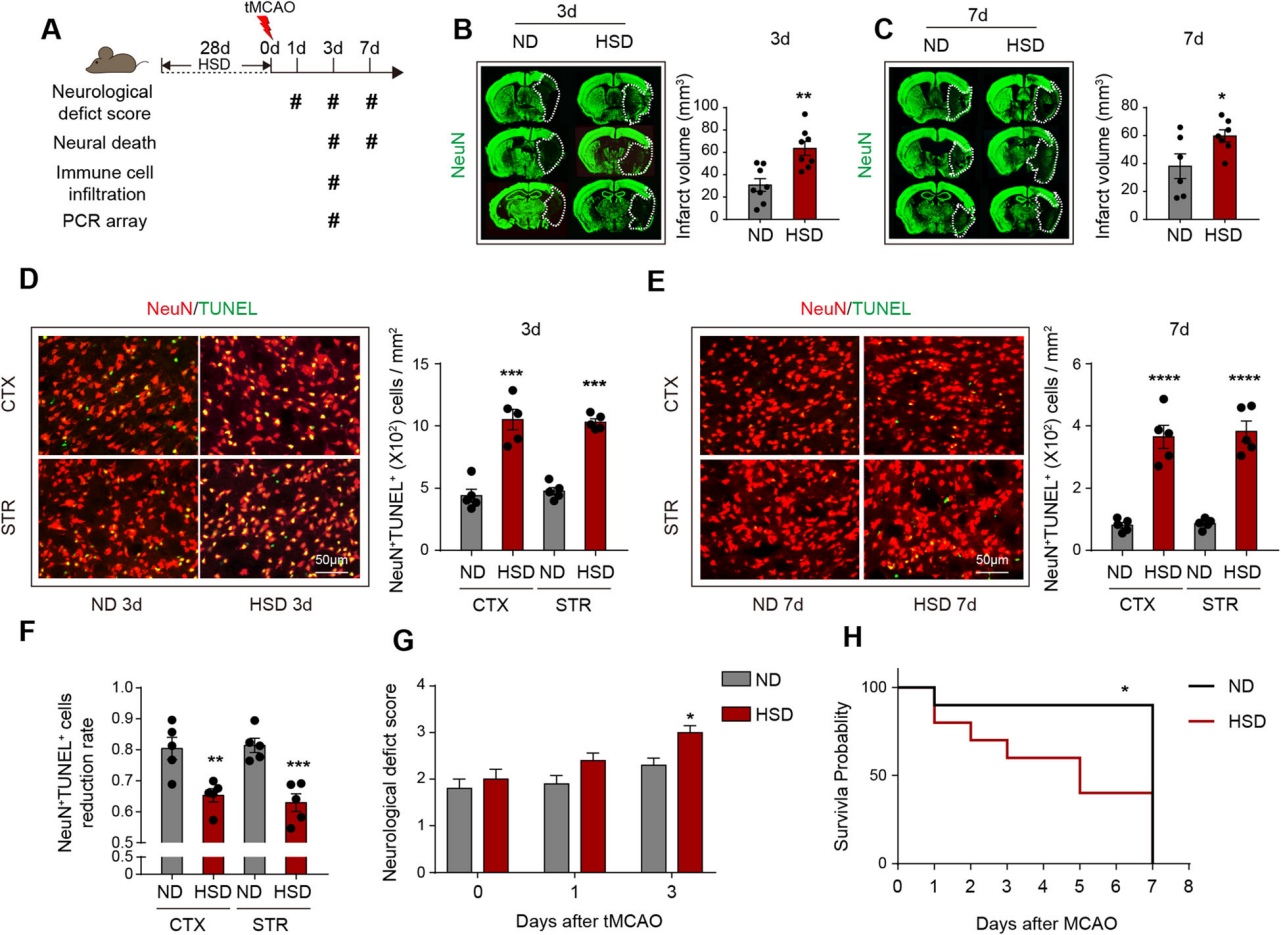
Excess salt intake exacerbates disease outcomes of ischemic stroke. C57BL/6 mice were fed with ND or HSD for 28d and were subjected to 60min of tMCAO. Animals were sacrificed at 3 days or 7 days after tMCAO. **a** The timeline of the in vivo experimental design. **b**, **c** Infarct volume of male mice was quantified in NeuN (green)-stained coronal sections at 3 days and 7 days in vivo. Dashed lines outline the infarct area. *N* = 6–8 mice per group, *P < 0.05 and **P < 0.01 versus ND group in *t* test. **d**, **e** Representative images demonstrating TUNEL (green) co-labeling with NeuN (red) in infarct penumbra at 3 days and 7 days after tMCAO in vivo. The number of NeuN^+^TUNEL^+^ neurons was quantified. *N* = 5 mice per group. ***P < 0.001 and ****P < 0.0001 versus ND group in *t* test. **f** The reduction rate of dead/dying neurons from 3 days–7 days after tMCAO was calculated in vivo. *N* = 5 mice per group, **P < 0.01 and ***P < 0.001 versus ND group in *t* test*.* **g** Neurological deficit score was assessed at 0–3 days after tMCAO. *N* = 10 per group, *P < 0.05 versus ND group in *t* test. **h** Survival of mice was recorded at 0–7 days after tMCAO. *N* = 10 mice per group. *P < 0.05 versus ND group in *t* test. ND normal diet, HSD high-salt diet, STR striatum, and CTX cortex

Healthy wild-type (WT) C57BL/6 male mice were fed with a high-salt diet (HSD) or normal diet (ND) for 28 days. Mice were then subjected to 60 min of transient middle cerebral artery occlusion (tMCAO) and sacrificed at 3 days or 7 days after cerebral ischemia (Fig. 1a). No significant alteration of salt concentration in the peripheral blood or bone marrow between ND mice and HSD mice was recorded (data not shown), which was consistent with the previous study [22]. Nevertheless, we observed increased expression of salt-inducible kinase1 (SIK1) in the ipsilateral brain of HSD mice at 3 days after tMCAO (Supplementary Figure 1A-B), which was a direct effect of sodium stimulation [23] and revealed the salinized microenvironment in stroke lesion of HSD mice. Consistent with a previous study [12, 24], HSD mice displayed increased lesion volume (Fig. 1b, c), detrimental neurological deficit (Fig. 1g), and poor survival rate (Fig. 1h). As assessed with immunostaining, we recorded accumulated dead neurons (NeuN^+^TUNEL^+^) in stroke penumbra (Fig. 1d, e). Strikingly, at 7 days after tMCAO, a 67% reduction in the number of dead neurons was observed (vs. 3 days) in HSD mice, which was less than that of mice fed with a normal diet (80%) (Fig. 1f). These results indicated that neurons in a high salinity environment suffered a processive injury and/or the injured neurons in HSD mice were not eliminated in time after stroke.

### Surplus salt intake amplifies post-stroke neural inflammation

**Fig. 2:**
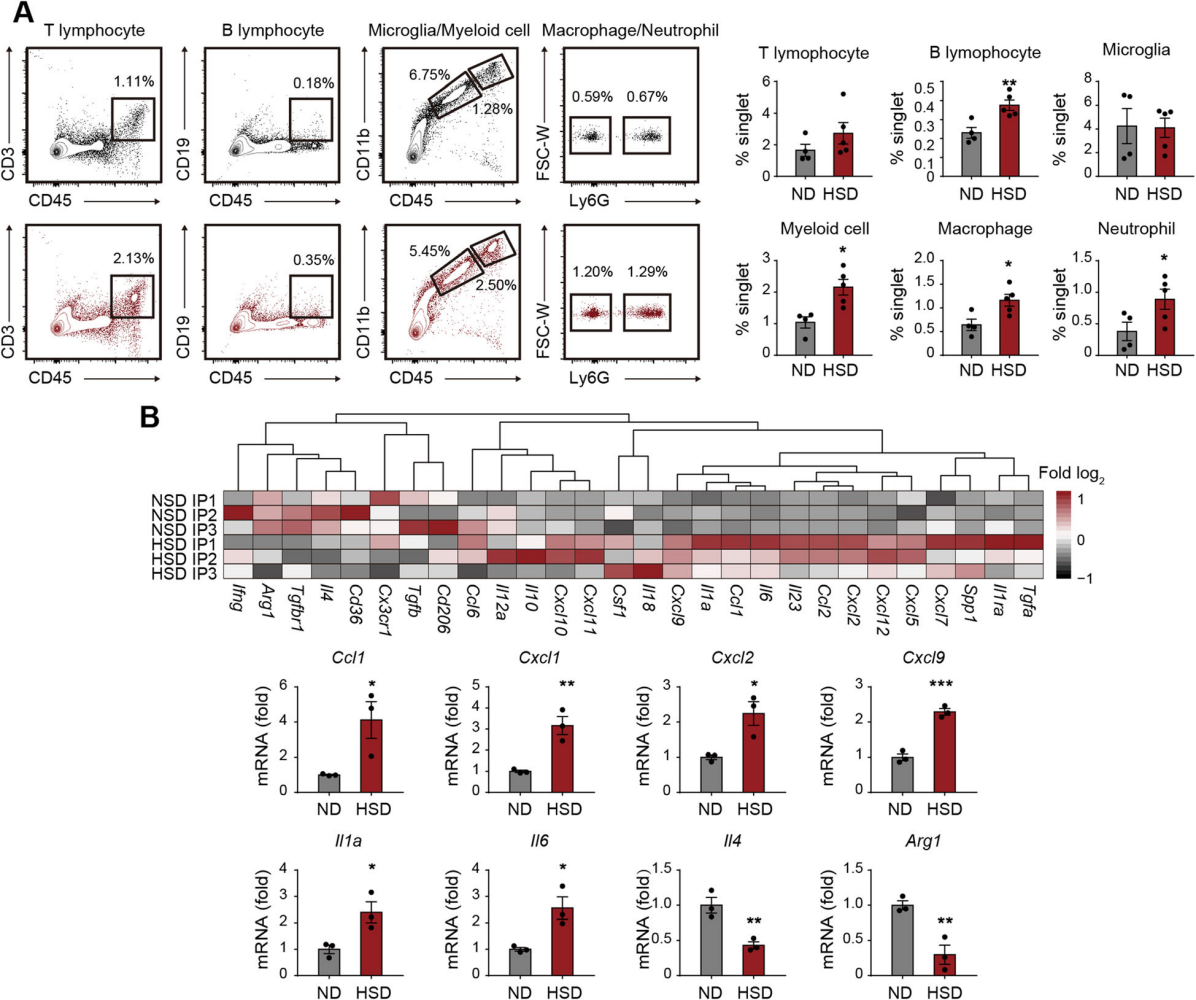
Surplus salt intake exacerbates post-stroke neural inflammation. **a** Leukocyte infiltration in the ischemic brain at 3 days after tMCAO was analyzed with flow cytometry in vivo. Representative flow plots showing infiltrated T lymphocytes (CD45^hi^CD3^+^), B lymphocytes (CD45^hi^CD19^+^), microglia (CD45^in^CD11b^+^), myeloid cells (CD45^hi^CD11b^+^), neutrophils (CD45^hi^CD11b^+^Ly6G^+^), and macrophages (CD45^hi^CD11b^+^Ly6G^-^) in ND and HSD brains, and the corresponding statistical analysis were displayed. *N* = 4 for ND and *N* = 5 mice for HSD group. *P < 0.05 and **P < 0.01 versus ND group in *t* test. **b** mRNA was extracted from the ipsilateral hemisphere of stroke mice at 3 days after tMCAO and subjected to RT-PCR. *N* = 3 mice per group. *P < 0.05, **P < 0.01, and ***P < 0.001 versus ND group in *t* test. IP ipsilateral

To examine the neural inflammatory status in HSD mice after tMCAO, we analyzed the infiltration of immune cells in stroke lesions using flow cytometry (Fig. 2a). We found that the percentages of T cells (CD45^+^CD3^+^), B cells (CD45^+^CD19^+^), neutrophils (CD45^hi^CD11b^+^Ly6G^+^), and macrophages (CD45^hi^CD11b^+^Ly6G^-^) among singlets increased in the ipsilateral hemisphere with ischemic stroke of HSD mice (Fig. 2a), while the composition of neutrophils (CD45^hi^CD11b^+^Ly6G^+^) and monocytes/macrophages (CD45^hi^CD11b^+^Ly6G^-^) in the peripheral blood and spleen remained comparable (Supplementary Figure 1C). Multiple proinflammatory cytokines and chemokines elevated in the ipsilateral brain of HSD mice (*Ccl1*, *Cxcl1*, *Cxcl2*, *Cxcl9*, *Il1a*, and *Il6*), while anti-inflammatory markers, including *Il4* and *Arg1*, decreased at the meantime (Fig. 2b and Supplementary Figure 1D). The results illustrated that post-stroke neural inflammation was amplified in HSD mice.

### Expression of inflammation resolution-associated molecules is downregulated in a high salinity environment

**Fig. 3:**
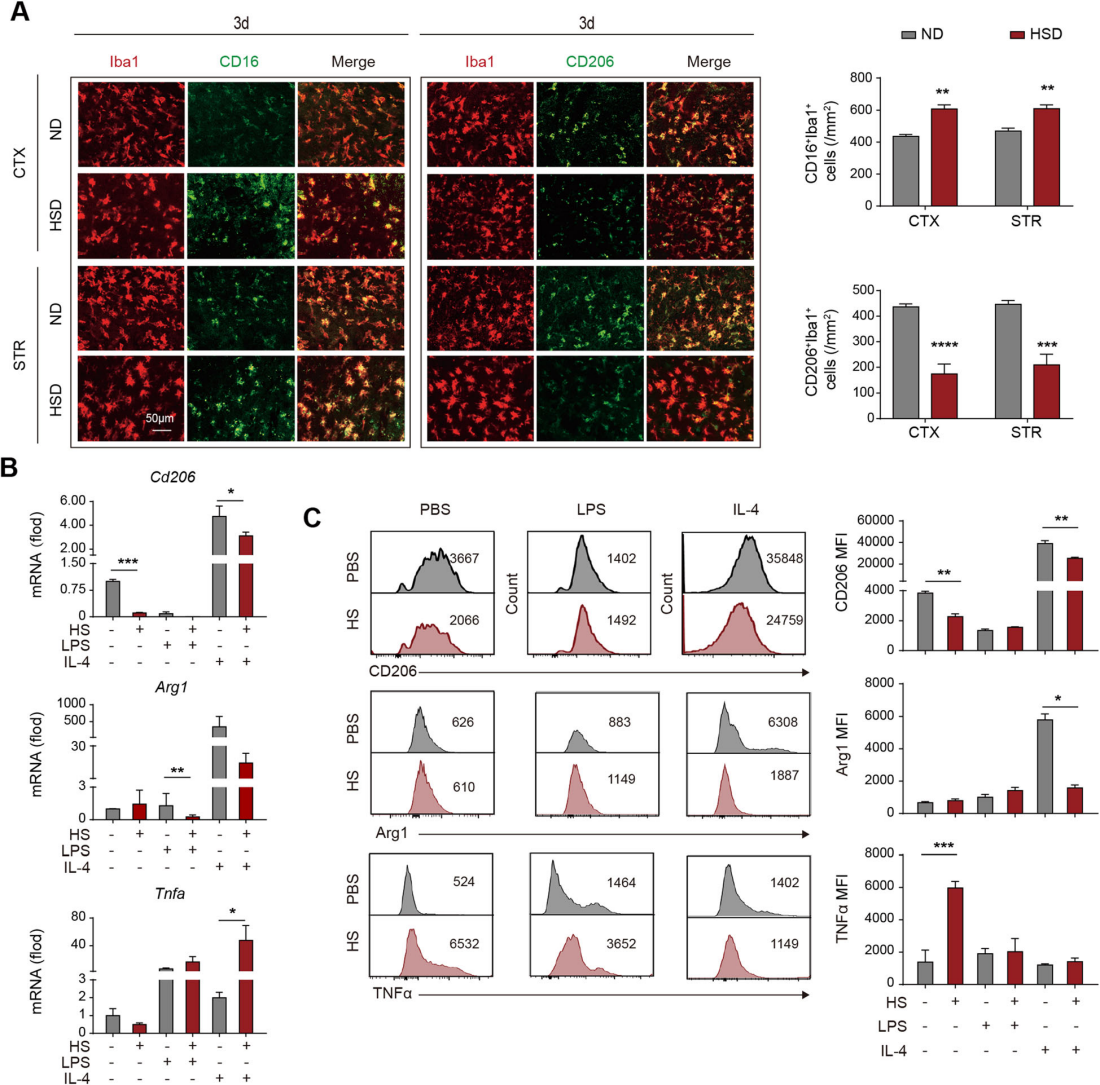
Expression of inflammation resolution-associated molecules in macrophages is downregulated in a high salinity environment. **a** Brain sections were collected from ND and HSD mice at 3 days after 60-min tMCAO. Expression of CD16 (green) or CD206 (green) in Iba1^+^ cells (a marker of microglia and macrophage) was analyzed with immunostaining in vivo. *N* = 3 mice per group. **P < 0.01, ***P < 0.001, and ****P < 0.0001 versus ND group in *t* test. **b**, **c** Bone marrow-derived primary cultured macrophages were treated with 40 mM of NaCl overnight at the presence of LPS (100 ng/ml) or IL-4 (20 ng/ml) in vitro. Expression of CD206, Arg1, and TNFα was assessed with RT-PCR (**b**) and flow cytometry (**c**). Comparable cell counts were analyzed among the PBS, HS, PBS+LPS, HS+LPS, PBS+IL-4, and HS+IL-4 groups. Data were collected from 3 independent experiments. *P < 0.05 and **P < 0.01 versus PBS group in *t* test. LPS lipopolysaccharide, STR striatum, CTX cortex

To testify the macrophages’ role in the drastic neural inflammation of HSD mice, we evaluated the inflammation resolution of these cells. Immunostaining revealed that the inflammation resolution-associated marker CD206 was downregulated in Iba1^+^ microglia/macrophages in the lesions of HSD mice at 3d after tMCAO (Fig. 3a). In contrast, the number of CD16 expressing Iba1^+^ microglia/macrophages was upregulated (Fig. 3a). To explore the impact of a high salinity environment on macrophages, we treated bone marrow-derived primary cultured macrophages with 40mM of NaCl overnight in the presence of LPS (100 ng/ml) or IL-4 (20 ng/ml). We recorded that a high concentration of NaCl alone reduced the expression of inflammation resolution marker CD206 as assessed with RT-PCR (Fig. 3b) and flow cytometry (Fig. 3c), and the tendency was more remarkable with the addition of LPS in the culture system (Fig. 3b, c). As was reported, IL-4 increased the expression of Arg1 and CD206 in primary cultured macrophages. Nevertheless, macrophages failed to respond to the IL-4 signaling in a high salinity environment (Fig. 3b, c). Macrophages pre-treated with NaCl, with or without the presence of IL-4, displayed elevated expression of TNFα (Fig. 3b, c). Thus, our data indicated that a high salinity environment undermined the anti-inflammatory or inflammation resolution property of macrophages.

### Efferocytosis of macrophages is impaired in a high salinity environment

**Fig. 4:**
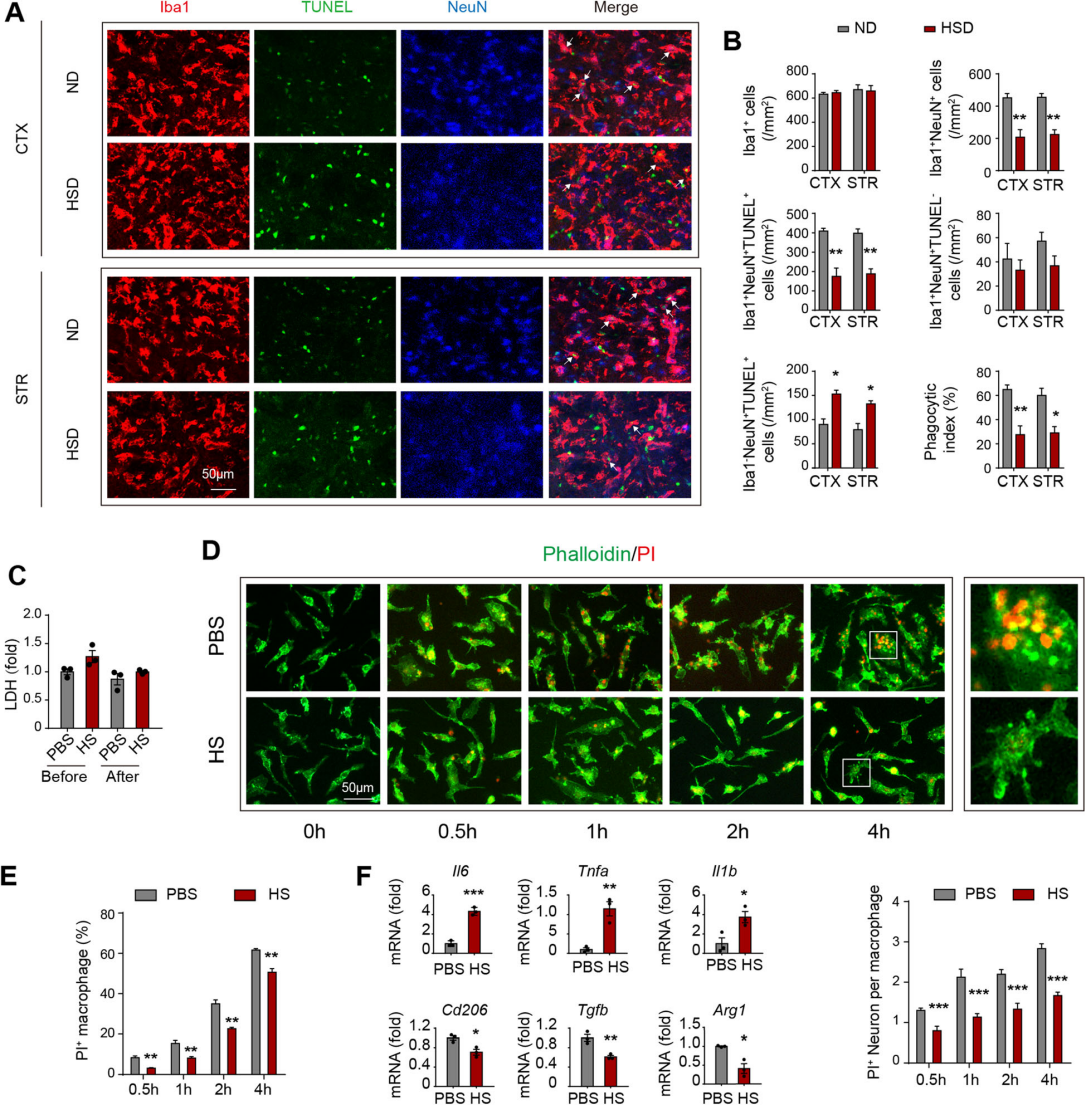
Efferocytosis of macrophages is impaired in a high salinity environment. **a**, **b** ND and HSD mice were subjected to 60min of tMCAO. Brain sections were collected at 3 days after cerebral ischemia. **a** Confocal microscopy analysis of NeuN (blue), TUNEL (green), and Iba1 (red) triple-staining in vivo. White arrows indicate microglia/macrophages that engulfed dead/dying neurons (Iba1^+^NeuN^+^TUNEL^+^). **b** Quantification of the total number of Iba1^+^ microglia/macrophages, Iba1^+^NeuN^+^ cells (microglia/macrophages that have engulfed neurons), Iba1^+^NeuN^+^TUNEL^+^ cells (microglia/macrophages that engulfed dead/dying neurons), Iba1^+^NeuN^+^TUNEL^-^ cells (microglia/macrophages that colocalize with TUNEL^-^ neurons), Iba1^-^NeuN^+^TUNEL^+^ cells (not engulfed dead neurons), and phagocytic index (the proportion of dead/dying neurons engulfed by microglia/macrophages) in ischemic areas in vivo. *N* = 3 mice per group. *P < 0.05 and **P < 0.01, versus ND group in *t* test. **c** Macrophage viability before and after 4h of neuron-efferocytosis was quantified by LDH-assay in vitro. Data were collected from 3 independent experiments. **d**, **e** Phagocytosis of PI^+^ dead/dying neurons by macrophages was evaluated in vitro with immunostaining (**d**) and flow cytometry (**e**). Engulfed dead/dying neurons (PI^+^) per macrophage (Phalloidin labeled) or the proportion of phagocytic macrophages (PI^+^) was quantified at the indicated time points. The right images enlarged the boxed area. Data were collected from three independent experiments. **P < 0.01 and ***P < 0.001 versus PBS group in *t* test. **f** Dead neurons were treated to macrophages at a ratio of neuron: macrophage = 5:1 in vitro. After 6h, expression of proinflammatory (*Tnfα*, *Il6*, and *Il1β*) and inflammatory resolving molecules (Arg1, CD206, TGFβ) in the efferocytic macrophages was analyzed with RT-PCR. Data represent three independent experiments performed in duplicate. *P < 0.05, **P < 0.01, and ***P < 0.001 versus PBS group in *t* test. STR striatum and CTX cortex

Efferocytosis represents an essential biological process for inflammation resolution mediated by macrophages. Therefore, we evaluated the impact of a high-salt environment on the phagocytic activities of macrophages. Clearance of dead/dying neurons was determined by detecting the neuronal marker NeuN within Iba1^+^ microglia/macrophages in stroke penumbra with confocal microscopy (Fig. 4a, b). Under the premise of a similar amount of Iba1^+^ cells (Fig. 4b), the number of Iba1^+^NeuN^+^ cells, which indicated the microglia/macrophages that had engulfed neurons, was reduced in HSD mice at 3 days after tMCAO compared to ND mice. Triple staining of Iba1/TUNEL/NeuN further revealed dampened phagocytosis of dead/dying neurons by microglia/macrophages in HSD mice, as the number of engulfed dead neurons (Iba1^+^NeuN^+^TUNEL^+^) decreased while the number of un-engulfed dead neurons (Iba1^-^NeuN^+^TUNEL^+^) increased. Very few Iba1^+^NeuN^+^TUNEL^-^ cells were observed in the stroke penumbra in both HSD and ND mice (Fig. 4a). Consistently, the phagocytic index, which was calculated as the proportion of dead/dying neurons engulfed by microglia/macrophages, was lower in HSD mice (Fig. 4b). We further evaluated the impact of high NaCl concentration on macrophage efferocytic activity upon encountering dead/dying neurons in vitro. Macrophages pre-exposed to the high salinity environment displayed reduced efferocytic capacity, as the engulfed dead/dying neurons per macrophage (Fig. 4d) or the proportion of phagocytic macrophages (PI^+^F4/80^+^) in high salinity environments (Fig. 4e) were lower than those in the control group after co-cultured for 0.5–4 h. However, no difference in cell viability between the two groups was observed (Fig. 4c). To estimate the cellular inflammation resolution capacity, we assessed the mRNA levels of the proinflammatory cytokine *Tnfα* and inflammatory resolving molecule *Arg1* at 6h after the onset of efferocytosis. Macrophages pre-treated with high NaCl displayed increased expression of *Tnfα* and reduced expression of *Arg1* compared with those treated with PBS (Fig. 4f). Overall, our results revealed that efferocytosis and the subsequent cellular inflammation resolution mediated by macrophages were impaired in high salinity environments.

### Excess salt downregulates TREM2 expression in macrophages and impairs inflammation resolution

**Fig. 5:**
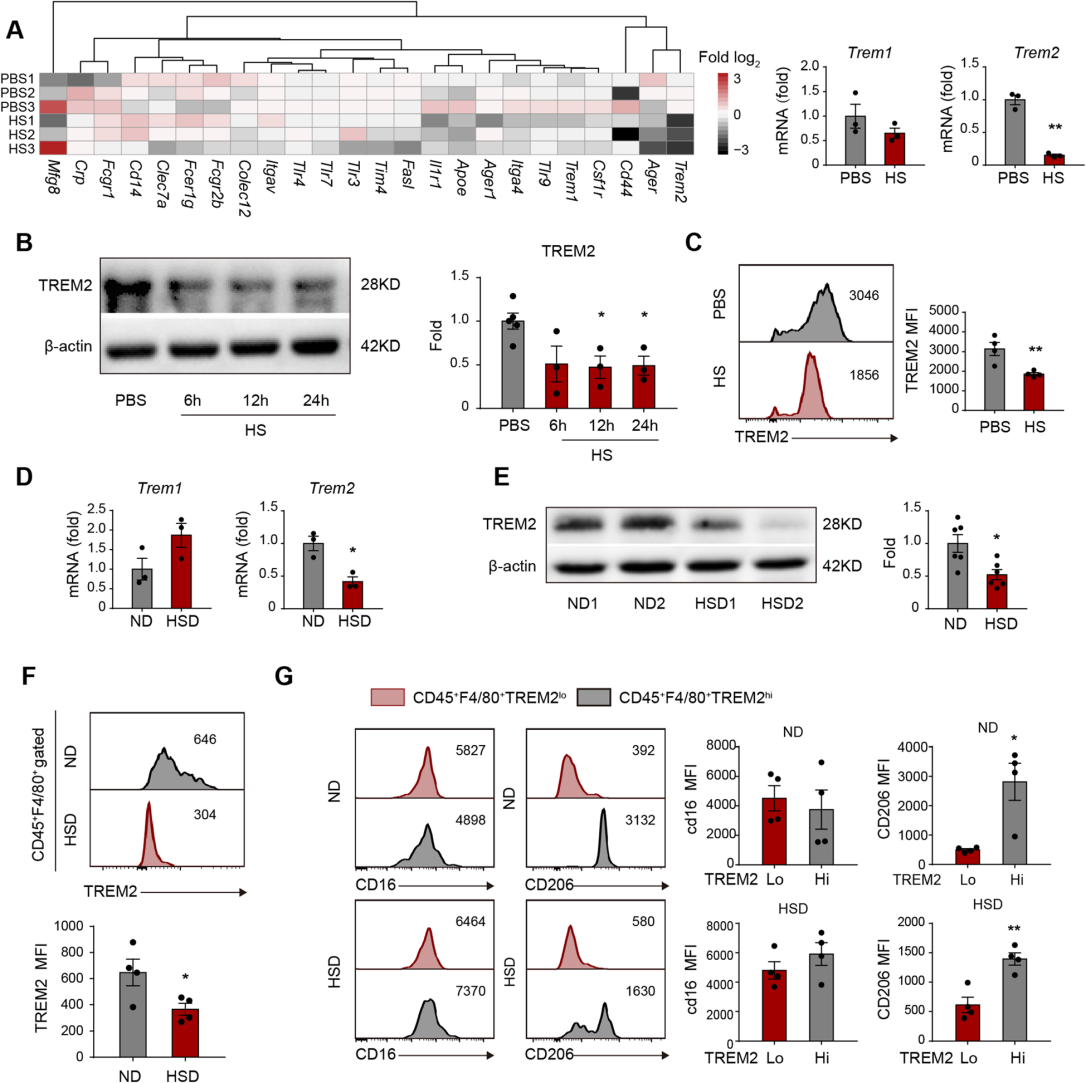
Excess salt downregulates efferocytic molecule TREM2 in macrophages and impairs their inflammation resolution functions. **a** Macrophages cultured in a high-salt environment were subjected to a PCR array to analyze the expression of efferocytosis-associated receptors in vitro. Data are displayed as fold change relative to macrophages from the PBS-treated group. Experiments were repeated three times. **P < 0.01 versus PBS group in *t* test. **b**, **c** Protein expression of TREM2 in macrophages after HS treatment was evaluated with western blot (**b**) and flow cytometry (**c**) in vitro. Experiments were repeated three times. *P < 0.05 versus PBS-treated group in *t* test. **d**–**g** ND and HSD mice were subjected to 60min of tMCAO. The brains were collected at 3 days after tMCAO. **d** mRNA expression of *Trem1* and *Trem2* in the ipsilateral hemisphere of stroke mice was analyzed with RT-PCR in vivo. CT value was normalized to that of ND mice. *N* = 3 mice per group. *P < 0.05 versus ND group in *t* test. **e** Protein expression of TREM2 in the ipsilateral hemisphere of stroke mice with western blot. *N* = 6 mice per group. *P < 0.05, versus ND group in *t* test. **f** In vivo, TREM2 protein expression in infiltrated macrophages (CD45^hi^CD11b^+^Ly6G^-^) of stroke mice was analyzed with flow cytometry. *N* = 4 mice per group. *P < 0.05 versus ND group in *t* test. **g** In vivo CD16 and CD206 expression in TREM2^lo^ and TREM2^hi^ macrophages (CD45^hi^CD11b^+^Ly6G^-^) in the ipsilateral hemisphere from ND and HSD mice was measured via flow cytometry. The mean fluorescence intensity (MFI) of CD16 or CD206 was quantified. *N* = 4 mice per group. *P < 0.05 and **P < 0.01 versus TREM2^lo^ macrophages in *t* test

To further investigate the underlying mechanism of attenuated macrophage efferocytosis, we evaluated the expression of phagocytosis-related receptors (PRRs) in macrophages treated with 40 mM of NaCl or an equal volume of PBS. We discovered that the high-salt environment downregulated the *Trem2* mRNA level, while the expression of other PRRs, including *Trem1* and *Tim4*, remained stable (Fig. 5a). Moreover, molecules downstream of TREM2 signaling were downregulated in high-salt concentrations, including *Arp2*, *Vav3*, and *Rac* [25, 26] (Supplementary Figure 2). We confirmed the downregulation of TREM2 in macrophages exposed to excess salt in vitro *by* western blot (Fig. 5b) and flow cytometric analysis (FACS) (Fig. 5c). We then examined TREM2 expression in vivo and found that the TREM2 mRNA (Fig. 5d) and protein levels (Fig. 5e, f) in the ipsilateral hemisphere of HSD mice were lower than those in ND mice at 3 days after tMCAO. Nevertheless, the level of TREM1 did not show a significant alteration in a high salinity environment (Supplementary Figure 3A-B). When investigating the relationship between TREM2 expression and inflammatory phenotype of macrophages with FACS, we found that macrophages with high TREM2 expression (CD45^+^F4/80^+^TREM2^hi^) displayed higher levels of the anti-inflammatory marker CD206 than those with low TREM2 expression (CD45^+^F4/80^+^TREM^lo^), while the CD16-MFI showed no difference between macrophages with high and low TREM2 expression in either HSD or ND mice (Fig. 5g).

### Decreased TREM2 expression is correlated with a proinflammatory property of circulating monocytes and detrimental stroke outcomes in AIS patients

**Fig. 6:**
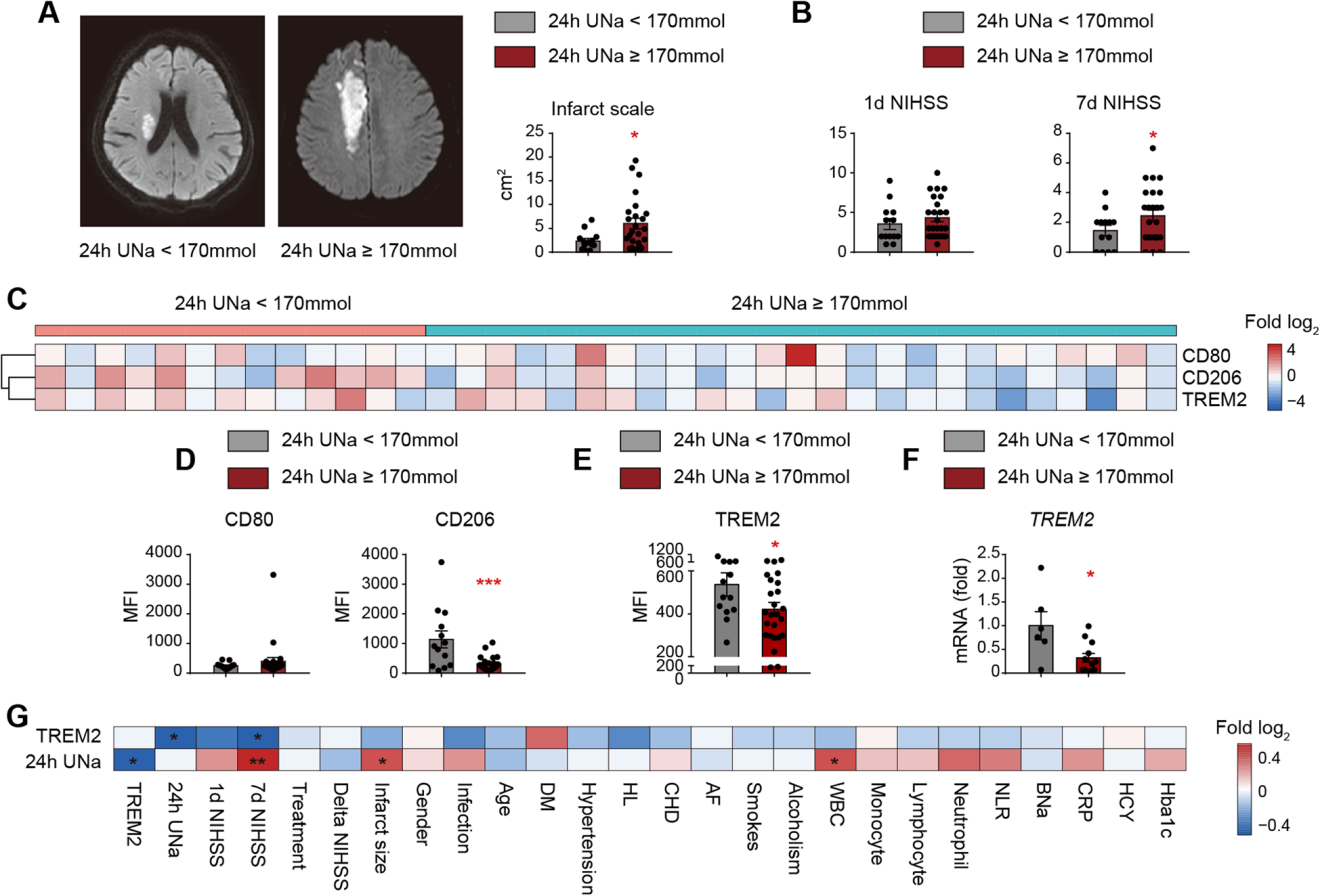
TREM2 expression is decreased in AIS patients with high-salt intake and is correlated with the proinflammatory property of circulating monocytes and detrimental stroke outcomes. AIS patient dietary salt intake was measured with a 24-h urine sodium (24h UNa) with a normal limit of 170 mmol. **a** Representative images of the MRI-DWI of AIS patients with normal or high urine sodium concentration. Comparison of infarct size of AIS patients with normal (*N* = 13) or high (*N* = 25) urine sodium concentration. *P < 0.05 by Student’s *t* test. **b** Comparison of 1-day NIHSS, 7-day NIHSS of AIS patients with normal (*N* = 13) or high (*N* = 25) urine sodium concentration. *P < 0.05 by Student’s *t* test*.* **c** Heat map showing TREM2, CD206, and CD80 protein expression in peripheral monocytes of AIS patients with normal (*N* = 13) or high (*N* = 25) urine sodium concentration using flow cytometry. The *function* of “scale” was applied for value normalization. **d** Comparison of CD206 and CD80 protein expression in peripheral monocytes of AIS patients with normal (*N* = 13) or high urine sodium concentration (*N* = 25) using flow cytometry. *P < 0.05 by Student’s *t* test. **e** Comparison of TREM2 protein expression in peripheral monocytes of AIS patients with normal (*N* = 13) or high urine sodium concentration (*N* = 25) using flow cytometry. *P < 0.05 by Student’s *t* test*.* **f** Comparison of *TREM2* mRNA expression in PBMCs of AIS patients with normal (*N* = 6) or high urine sodium concentration (*N* = 12). *P < 0.05 by Student’s *t* test. **g** Correlation of clinic parameters, the protein expression of TREM2 and 24h UNa was assessed with Spearman correlation analysis. *P < 0.05, **P < 0.01. DWI diffusion-weighted imaging, DM diabetes mellitus, HL hyperlipidemia, CHD coronary heart disease, AF atrial fibrillation, WBC white blood cell, NLR neutrophil-to-lymphocyte ratio, BNa blood sodium, CRP C-reactive protein, HCY homocysteine, and Hba1c glycated hemoglobin

We then tested the TREM2 level in monocytes of AIS patients and evaluated the relationship between TREM2 expression and stroke outcomes. AIS patients’ dietary salt intake was measured with a 24-h urine sodium with a normal limit of 170 mmol [27]. Thereafter, we found that patients with high urine sodium concentrations had larger infarct sizes (Fig. 6a) and higher NIHSS scores (Fig. 6b) than those with normal urine sodium concentrations. To assess the impact of excessive salt on the phenotypic shift of circulating monocytes in AIS patients during the acute phase (0–3 days after disease onset), we analyzed the expression of the proinflammatory marker CD80 and the anti-inflammatory marker CD206 [28–30] in monocytes (CD11b^+^CD14^+^) of patient peripheral blood with FACS. Detailed gating strategy is displayed in Supplementary Figure 4. As expected, monocytes from stroke patients with high urine sodium concentration expressed less CD206 than normal monocytes from diet stroke patients, while no differential expression of CD80 was recorded (Fig. 6c, d and Supplementary Figure 5). TREM2 expression in monocytes was downregulated in stroke patients with high urine sodium concentration compared with those with normal diets using FACS (Fig. 6c, e and Supplementary Figure 5). Moreover, we found that the *TREM2* mRNA level decreased in the peripheral blood mononuclear cells (PBMC) of patients with high urine sodium concentration (Fig. 6f). At the same time, the expression of other PRRs remained to be stable (Supplementary Figure 6). Since PRRs are mainly expressed in monocytes in PBMC [31], our data indicated that a high salinity environment specifically downregulated *TREM2* expression in monocytes of AIS patients. Through *Spearman correlation analysis*, we determined that the CD206 MFI of peripheral blood monocytes showed a significant positive correlation with TREM2 MFI in stroke patients, while the CD80 MFI showed a negative correlation with the TREM2 MFI (Supplementary Figure 7), which was consistent with our data in animal models. Interestingly, we found that TREM2 expression in the circulating monocytes of AIS patients was negatively correlated with a 24-h urine excretion (Fig. 6g and Supplementary Figure 7), while decreased TREM2 level of macrophages was associated with increased NIHSS scores (Fig. 6g and Supplementary Figure 7). The results indicated that TREM2 expression in monocytes/macrophages favored efferocytosis and the subsequent inflammation resolution after ischemic stroke.

### Enhancement of TREM2 signaling restores the efferocytic capacity and cellular inflammation resolution of macrophages in the high salinity environment

**Fig. 7:**
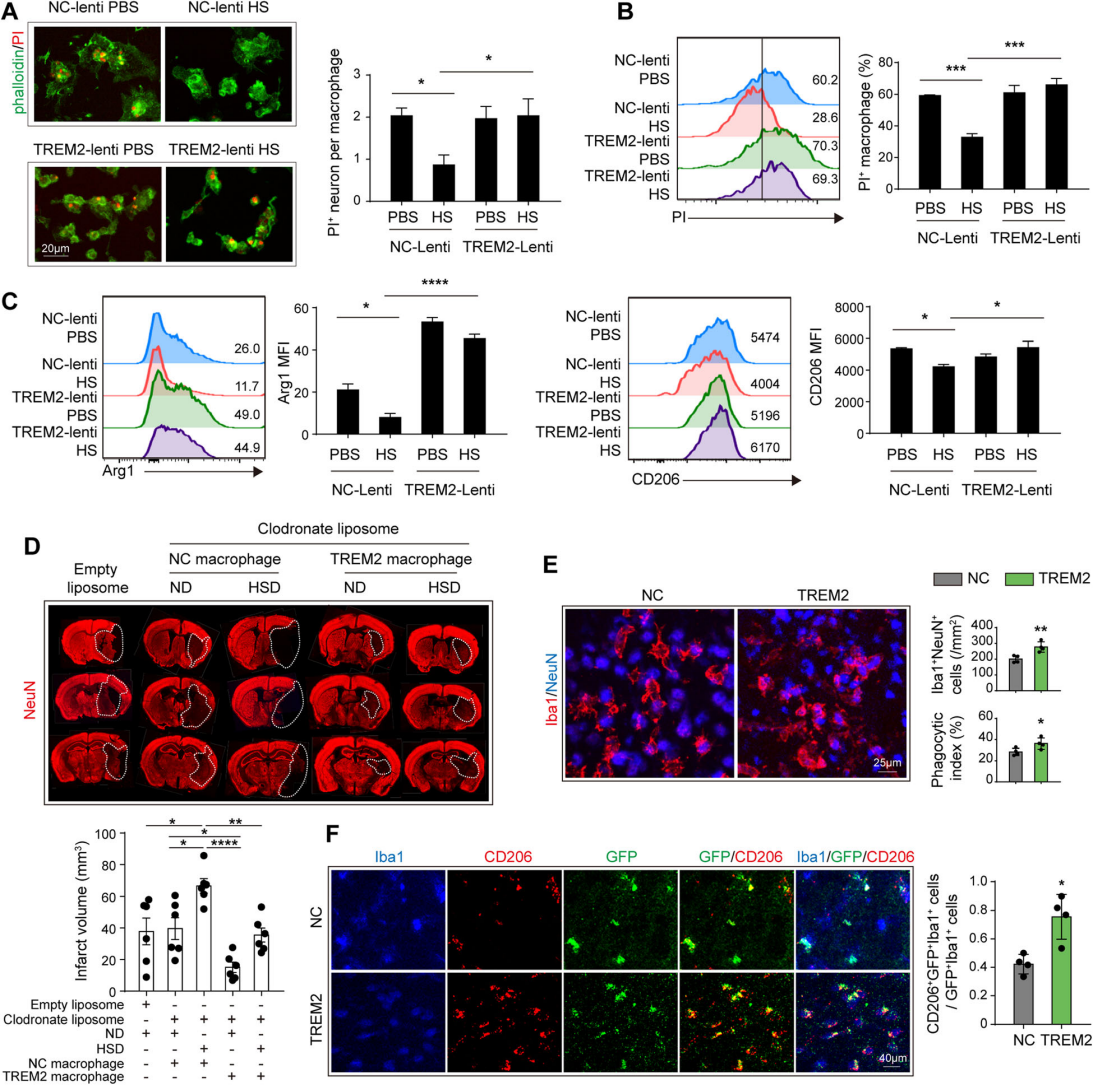
Enhancement of TREM2 signaling restores the efferocytic capacity and cellular inflammation resolution of macrophages in a high salinity environment. Bone marrow-derived primary cultured macrophages were infected with lentiviral vectors carrying TREM2-GFP cDNA (TREM2-Lenti) or control lentivirus carrying GFP only (NC-Lenti). Macrophages were subjected to analysis at 2 days after infection. **a**, **b** Transfected macrophages were treated in vitro with HS or PBS overnight and then subjected to efferocytic analysis with PI^+^ dead/dying neurons. The efferocytic efficiency of macrophages was evaluated with immunostaining (**a**) and flow cytometry (**b**). Engulfed dead/dying neurons (PI^+^Phalloidin^+^) per macrophage or the proportion of phagocytic macrophages (PI^+^F4/80^+^) was calculated at the corresponding time points. Data were collected from three independent experiments. *P < 0.05 and ***P < 0.001 versus NC-Lenti PBS group in *one-way ANOVA.* **c** In vitro protein expression of Arg1 and CD206 in transfected macrophages with HS or PBS treatment overnight was analyzed with flow cytometry. *P < 0.05 versus NC-Lenti PBS group in *one-way ANOVA.* **d**, **e** C57BL/6 mice with HSD or ND were first injected with clodronate liposomes to deplete monocytes/macrophages. Mice were then subjected to 60 min of tMCAO. Immediately after reperfusion, a single dose of 2x10^6^ TREM2-overexpressing macrophages or NC macrophages were intravenously transferred in vivo. **d** The in vivo infarct volume of male mice was quantified on NeuN (red)-stained coronal sections at 3 days. Dashed lines outline the infarct area. *N* = 6 mice per group, *P < 0.05, **P < 0.01, and ****P < 0.0001, *one-way ANOVA*. **e** Confocal microscopy analysis of NeuN (blue) and Iba1 (red) double-staining in HSD mice. Quantification of the total number of Iba1^+^NeuN^+^ cells (microglia/macrophages that have engulfed neurons) and phagocytic index (the proportion of neurons engulfed by microglia/macrophages) in ischemic areas of HSD mice. *N* = 4 mice per group. *P < 0.05 and **P < 0.01 versus NC macrophage group in *t* test. **f** In vivo CD206 (red) expression in Iba1^+^GFP^+^ cells was analyzed via immunostaining. *N* = 4 mice per group. *P < 0.05 versus NC macrophage group in *t* test

TREM2 is a vital functional molecule implicated in the phagocytosis activity of macrophages. The efferocytosis capacity of macrophages plays a decisive role in inflammation resolution after stroke and affects the disease outcomes. Therefore, we hypothesized that enhancing TREM2 signaling in macrophages could restore their efferocytic capacity and promote inflammation resolution. Macrophages were infected with lent viral vectors carrying TREM2 cDNA or empty vector (NC) for 2 days before treatment. Efficacy of transfection was confirmed with flow cytometry (Supplementary Figure 8A) and western blot (Supplementary Figure 8B). Transfected macrophages were treated with or without the addition of NaCl (40mM) and incubated with PI-labeled post-OGD neurons. Gratifyingly, TREM2 overexpression in macrophages exposed to excess salt restored the efferocytic capacity, as the engulfed dead/dying neurons per macrophage (Fig. 7a), or the proportion of phagocytic macrophages (PI^+^F4/80^+^) (Fig. 7b) recovered to the levels of those treated with PBS at 1h after co-culture. Moreover, at 24 h after co-culture, protein levels of CD206 and Arg1 in TREM2-overexpressing macrophages treated with a high concentration of NaCl resembled those treated with PBS (Fig. 7c). To further evaluate the effect of TREM2-overexpressing macrophages on high-salt-afforded neuroinflammation after ischemic stroke, mice with HSD or ND were first injected with clodronate liposomes to deplete monocytes/macrophages (Supplementary figure 9). Mice were then subjected to 60 min of tMCAO. Immediately after reperfusion, a single dose of 2x10^6^ TREM2-overexpressing macrophages or NC macrophages were intravenously transferred. In ND mice, we recorded that transfer of TREM2-overexpressing macrophages reduced the infarct volume compared with those transferred with NC macrophages (Fig. 7d). In addition, transfer of TREM2-overexpressing macrophages offered protection to HSD mice. Infarct volume decreased in HSD mice transferred with TREM2-overexpressing macrophages. We recorded restored efferocytic capacity of the infiltrated macrophages in HSD mice transferred with TREM2-overexpressing macrophages, as the number of phagocytic microglia/macrophages (Iba1^+^NeuN^+^) and phagocytic index in ischemic lesions increased compared with those transferred with NC macrophages (Fig. 7e). The percentage of CD206 expressing Iba1^+^GFP^+^ macrophages in the lesions was higher in HSD mice transferred with TREM2-overexpressing macrophages (Fig. 7f). Our data revealed that enhancing TREM2 signaling could restore the efferocytic capacity and cellular inflammation resolution of macrophages that were damaged in the high sodium microenvironment.

## Discussion

The current study demonstrates that a microenvironment with an excess salt concentration could impair the inflammation resolution property of macrophages after ischemic stroke. Mechanistically, excessive salt downregulates TREM2 expression in macrophages associated with decreased efferocytic capacity, excessive neural inflammation, and exacerbated stroke outcomes.

Given the diverse and broad effect of sodium ions, histocytes and the urinary system keep sodium in strict equilibrium. For being fed with the high-salt diet for as long as 4 weeks, sodium concentration in the peripheral blood and interstitial fluid of HSD mice still remained stable. It remains to be demonstrated whether smaller changes in Na concentration have similar effects on macrophage function. The transient fluctuation of sodium concentration along with daily meals, which are insistently repeated during the high-salt diet, could be sufficient to exert effective impacts. In accordance, we recorded that circulating monocytes in stroke patients with high-salt consumption displayed proinflammatory inclination compared with those in patients with appropriate salt intake, which was presented in culture macrophages directly exposed to a high-salt environment in vitro. Further, in the arena of stroke lesion, ongoing neural inflammation and local metabolic disturbance could facilitate sodium accumulation. Excessive expression of SIK1 in stroke lesion of HSD mice revealed the salinized microenvironment encountered by the infiltrated macrophages. To explore the molecular mechanisms of functional alterations of macrophages when encountering sodium stimulation, bone marrow-derived macrophages were cultured and threatened with high sodium treatment. Although the in vitro culture system failed to perfectly reproduce the complicated and dynamic pathophysiological process in vivo, we recorded distinctive proinflammatory activations in high-salt-treated macrophages, which were further verified in HSD stroke mice and AIS patients.

It has been reported that the HSD could promote BBB injury after ischemic stroke [12]. Consistently, we recorded that increased infiltration of multiple leukocytes, including macrophages, neutrophils, T lymphocytes, and B lymphocytes, in the stroke lesions of HSD mice at 3 days after stroke, could be attributed to the exacerbated BBB damage. It was found that surplus dietary salt directed macrophages/microglia towards the classical activated “M1” phenotype, which further exacerbated stroke outcomes [24]. In accordance, our data indicated that the inflammation resolution property of macrophages was downregulated by excess salt, which led to the postponed recovery of stroke lesions.

Efferocytosis represents an essential process of inflammation resolution [32, 33]. Elimination of the dead or injured components within stroke lesion arrests amplification of neural inflammation. We demonstrated that the efferocytic capacity, together with the subsequent cellular inflammation resolution of macrophages, was impaired in the high salinity environment, which could be a rational explanation for accumulated dead cells in the stroke penumbra. It has been demonstrated that the function of TREM2 is indispensable for phagocytic activities of microglia and macrophages [34]. Our data indicated that TREM2 was downregulated in macrophages in the high salinity environment. Decreased TREM2 expression was correlated with robust post-stroke neural inflammation and exacerbated stroke outcomes, which indicated that inhibition of TREM2 signaling in macrophages was the potential mechanism involved in the detrimental impact of the high-salt microenvironment.

It has been recognized that HSD is a crucial risk factor for ischemic stroke. Restriction of dietary salt intake serves as an efficient and practical method for preventing new vascular events. Nevertheless, no niched therapy that targets the already impaired inflammation resolution property of macrophages in the high-salt environment has been reported. In our study, utilization of TREM2-overexpressing macrophages offered neuroprotection to HSD mice. In addition, overexpression of TREM2 restored the efferocytic capacity and cellular inflammation resolution of macrophages in a high salinity environment. The data encourage further research on the therapeutic potential of enhancing TREM2 signaling in patients with ischemic stroke, especially those with high-salt intake.

## Conclusions

Conclusively, HSD aggravates ischemic stroke outcomes by exacerbating neural inflammation, which is associated with the impaired inflammation resolution property of macrophages. TREM2 expression in macrophages is downregulated by high-salt environments and enhancing TREM2 signaling could restore the efferocytic capacity and cellular inflammation resolution of macrophages. Further study on the value of TREM2 signaling as a therapeutic target in AIS is warranted.

## Supplementary Information

**Additional file 1: Table S1.** Clinic characteristics of the whole cohort. **Table S2.** Primers used in the study. **Figure S1.** Comparison of inflammatory mediator expression in peripheral blood and contralateral brains of ND and HSD mice. **Figure S2.** Excess salt downregulates efferocytic molecules in macrophages. **Figure S3.** Impact of high salt on TREM1 expression in primary culture macrophage and ischemic brain. **Figure S4.** Gating strategy in flow cytometric analysis of TREM2, CD80, and CD206 expression in monocytes of AIS patients. **Figure S5.** Representative images of TREM2, CD206, and CD80 expression in peripheral monocyte of AIS patients with normal or high urine sodium concentration. **Figure S6.** PRRs mRNA expression remained no different other than *TREM2* in AIS patients with high salt intake. **Figure S7.** Spearman correlation analysis of TREM2 expression, monocyte phenotypic marker, AIS outcomes and clinic parameters. **Figure S8.** Validation of TREM2 overexpression efficacy in primary cultured macrophages. **Figure S9.** Circulating macrophages and monocytes were depleted by clodronate liposome treatment.

## Data Availability

The datasets used and/or analyzed during the current study are available from the corresponding author on reasonable request.
