# Supplementary material for: High-salt diet downregulates TREM2 expression and blunts efferocytosis of macrophages after acute ischemic stroke
Source: J Neuroinflammation. 2021 Apr 12;18:90. doi: 10.1186/s12974-021-02144-9 (PMC8040220; doi:10.1186/s12974-021-02144-9)
Supplement: Supplementary file 1 — Additional file 1: Table S1. Clinic characteristics of the whole cohort. Table S2. Primers used in the study. Figure S1. Comparison of inflammatory mediator expression in peripheral blood and contralateral brains of ND and HSD mice. Figure S2. Excess salt downregulates efferocytic molecules in macrophages. Figure S3. Impact of high salt on TREM1 expression in primary culture macrophage and ischemic brain. Figure S4. Gating strategy in flow cytometric analysis of TREM2, CD80, and CD206 expression in monocytes of AIS patients. Figure S5. Representative images of TREM2, CD206, and CD80 expression in peripheral monocyte of AIS patients with normal or high urine sodium concentration. Figure S6. PRRs mRNA expression remained no different other than TREM2 in AIS patients with high salt intake. Figure S7. Spearman correlation analysis of TREM2 expression, monocyte phenotypic marker, AIS outcomes and clinic parameters. Figure S8. Validation of TREM2 overexpression efficacy in primary cultured macrophages. Figure S9. Circulating macrophages and monocytes were depleted by clodronate liposome treatment. [file 12974_2021_2144_MOESM1_ESM.docx]

**eSupplementary Materials**

**Supplementary Tables**

| **Supplementary Table 1. Clinic characteristics of the whole cohort.** | | | | |  |  |  |  |  |
| --- | --- | --- | --- | --- | --- | --- | --- | --- | --- |
|  | **Cohort 1** | | | |  | **Cohort 2** | | | |
| **Clinical characteristics** | **24h UNa < 170mM (*N*=13)** | **24h UNa ≥ 170mM (*N*=25**) | **Total (*N*=38)** | ***P*** |  | **24h UNa < 170mM (*N*=6)** | **24h UNa ≥ 170mM (*N*=12**) | **Total (*N*=18)** | ***P*** |
| **Demographics** |  |  |  |  |  |  |  |  |  |
| Age, y, median (quartiles) | 66.0 (59.0-76.0) | 57.0 (53.0-68.0) | 61.5 (54.0-71.0) | 0.25136 |  | 64.0 (61.2-69.8) | 56.5 (47.0-68.0) | 61.0 (53.0-68.0) | 0.09249 |
| Females gender, *N* (%) | 4 (31%) | 6 (24%) | 10 (26%) | 0.70920 |  | 5 (83.3%) | 9 (75.0%) | 14 (77.8%) | 1.00000 |
| DM, *N* (%) | 2 (15.4%) | 5 (20%) | 7 (18.4%) | 1.00000 |  | 2 (33.3%) | 2 (16.7%) | 4 (22.2%) | 0.56860 |
| Hypertension, *N* (%) | 7 (53.8%) | 10 (40%) | 17 (44.7%) | 0.50170 |  | 5 (83.3%) | 4 (33.3%) | 9 (50.0%) | 0.13120 |
| Hyperlipidemia, *N* (%) | 1 (7.69%) | 5 (20%) | 6 (5.25%) | 1.00000 |  | 1 (16.7%) | 2 (16.7%) | 3 (16.7%) | 1.00000 |
| Coronary heart disease, *N* (%) | 1 (7.69%) | 2 (8%) | 3 (7.89%) | 1.00000 |  | 1 (16.7%) | 1 (8.3%) | 2 (11.1%) | 1.00000 |
| Atrial fibrillation, *N* (%) | 1 (7.69%) | 1 (4%) | 2 (5.26%) | 0.52380 |  | 0 (0%) | 0 (0%) | 0 (0%) | 1.00000 |
| Smoke, *N* (%) | 3 (23%) | 9 (36%) | 12 (31.6%) | 0.71480 |  | 2 (33.3%) | 2 (16.7%) | 4 (22.2%) | 0.56860 |
| Alcoholism, *N* (%) | 2 (15%) | 3 (12%) | 5 (13%) | 0.63090 |  | 1 (16.7%) | 1 (8.3%) | 2 (11.1%) | 1.00000 |
| 1d NIHSS, median (quartiles) | 2.0 (2.0-5.0) | 3.0 (2.0-6.0) | 3.0 (2.0-5.0) | 0.18055 |  | 2.0 (2.0-2.0) | 5.5 (3.0-7.2) | 4.0 (2.0-6.0) | 0.00305 |
| 7d NIHSS, median (quartiles) | 2.0 (0-2.0) | 2.0 (1.0-4.0) | 2.0 (1.0-3.0) | 0.03266 |  | 0.5 (0-1.0) | 4.0 (1.8-5.0) | 2.0 (1.0-4.0) | 0.00005 |
| delta NIHSS, median (quartiles) | 1.0 (1.0-3.0) | 1.0 (1.0-2.0) | 1.0 (1.0-2.8) | 0.61240 |  | 1.0 (1.0 -1.8) | 2.0 (1.0-2.2) | 1.5 (1.0-2.0) | 0.70656 |
| **Laboratory Variables, Median (quartiles)** | | |  |  |  |  |  |  |  |
| 24h urine sodium (mmol/24H) | 141.0 (99.6-151.2) | 264.0 (215.6-302.4) | 212.8 (154.7-276.7) | 0.00000 |  | 106.1 (100.7-150.8) | 286.1 (230.5-351) | 225.5 (165.9-325.2) | 0.00003 |
| Blood sodium (mmol/L) | 142.0 (139-142.3) | 141.8 (140-142.5) | 142.0 (140.0-142.5) | 0.83714 |  | 141.9 (139-143.1) | 142.0 (140.8-142) | 142.0 (140.2-142.0) | 0.78588 |
| C reaction protein (mg/L) | 1.9 (1.4-10.9) | 3 (1.3-11.8) | 2.8 (1.3-11.6) | 0.50510 |  | 9.3 (3.9-12) | 1.6 (1.2-8.2) | 2.8 (1.3-11.6) | 0.43531 |
| Homocysteine (μmol/L) | 12.42 (11.0-15.6) | 17.8 (11.2-24.7) | 14.3 (11.1-19.2) | 0.01690 |  | 11.8 (11.2-13.7) | 10.1 (7.3-12) | 10.6 (8.8-13.7) | 0.85092 |
| Hbac1 (%) | 5.7 (5.5-6) | 5.8 (5.5-6.3) | 5.8 (5.5-6.2) | 0.14408 |  | 5.8 (5.6-6.2) | 5.8 (5.4-6.1) | 5.8 (5.5-6.2) | 0.83872 |
| WBC (X10^9^/L) | 6.3 (6.0-7.4) | 8.7 (7.7-10.1) | 7.9 (6.4-10.1) | 0.21805 |  | 6.0 (5.8-7.1) | 8.3 (7.1-9.7) | 7.6 (6.1-9.3) | 0.08688 |
| Neutrophil (X10^9^/L) | 4.4 (3.5-4.8) | 5.6 (4.5-7.8) | 4.9 (3.9-7.6) | 0.28585 |  | 3.6 (3.2-4.2) | 4.9 (3.8-6.7) | 4.5 (3.4-6.2) | 0.20759 |
| Lymphocyte (X10^9^/L) | 1.5 (1.2-2.0) | 2.0 (1.4-2.9) | 1.7 (1.3-2.7) | 0.37807 |  | 1.7 (1.4-2.3) | 2.1 (1.4-3.6) | 2.2 (1.6-3.2) | 0.42802 |
| Monocyte (X10^9^/L) | 0.5 (0.4-0.5) | 0.5 (0.3-0.6) | 0.5 (0.4-0.6) | 0.72389 |  | 0.5 (0.4-0.6) | 0.5 (0.3-0.6) | 0.5 (0.4-0.6) | 1.00000 |
| NLR | 2.7 (2.1-3.3) | 2.4 (1.7-4.6) | 2.6 (1.9-4.2) | 0.50500 |  | 2.4 (1.9-2.7) | 2.1 (1.5-3.5) | 2.2 (1.6-3.2) | 0.54510 |

DM, diabetic mellitus; Hba1c, glycated hemoglobin; WBC, white blood cell. ND, normal diet, defied as 24h urine sodium < 170mmol; HSD, high salt diet, defied as 24h urine sodium ≥ 170mmol; NLR, neutrophil-to-lymphocyte ratio. *P* values were obtained by *Student’s t-test* and *chi-square test*.

| **Supplementary Table 2. Primers used in the study.** | | |
| --- | --- | --- |
| **Gene** | **Forwards primer** | **Reverse primer** |
| **Mus musculus** | | |
| *Msn* | TAAAGTCGTGGCCCGTTAGC | TGACACGCACACTGATCGTC |
| *Rhoa* | CCGTCGGTTCTCTCCATAGC | TCTCAGATGCAAGGCTCAAGG |
| *Arp2* | ACCGATATGGACAGCCTTCTG | ACCACGTAGCAGAGGTAGGT |
| *Myd88* | TAGGACAAACGCCGGAACTT | ATGCGGCGACACCTTTTCTC |
| *Itgam* | TTGCCTCGAGGGCAGAGG | CATTCACGTCTCCCAGCACT |
| *Syk* | AGCAGGAAACCTCCACTTGC | GTGTATGGAGAAGTACCTTCCTGT |
| *Tnfa* | CTGAACTTCGGGGTGATCGG | GGCTTGTCACTCGAATTTTGAGA |
| *Arp3* | GAGCCTTTCATTTCCTCAGGCT | CGTGGATGTTGTCCTTGTTCT |
| *C3* | ATAAAGAGCCAGCGGCTACA | GGGAGTAATGATGGAATACATGGGG |
| *Alkbh5* | TGACTGTGCTCAGTGGGTATG | TTCCAATCGCGGTGCATCTA |
| *Itga4* | AACCGGGCACTCCTACAAC | CACCACCGAGTAGCCAAACAG |
| *Pten* | TCCCAGACATGACAGCCATC | TGCTTTGAATCCAAAAACCTTACT |
| *Mapk14* | AAGACTCGTTGGAACCCCAG | TCCAGTAGGTCGACAGCCAG |
| *Ager* | CACGAGGATGAGGGCACCTA | CTCATCGCCGGTTTCTGTGA |
| *Tlr9* | GAGAGACCCTGGTGTGGAAC | CCTTCGACGGAGAACCATGT |
| *Tlr4* | AATCCCTGCATAGAGGTAGTTCC | ATCCAGCCACTGAAGTTCTGA |
| *Trem1* | AGTCGTTGGAGCTGAGCTTG | GGCAGCTTTGACTTCTGAGAC |
| *Fcgr1* | TCGGTGGGGAAGTGGTTAATG | ACTGGCCTCTGGGATGCTAT |
| *Pros1* | CCTCTCAGCAATGAGGGTCC | CACGCTCTTTGGACAAGAAGTT |
| *Tlr7* | ATGTGGACACGGAAGAGACAA | ACCATCGAAACCCAAAGACTC |
| *Vav3* | GTGGCTCATCCACAGCAAGG | TCTTCAAACAGAGAAACTGGGACA |
| *Fasl* | TCCGTGAGTTCACCAACCAA | TGAGTGGGGGTTCCCTGTTA |
| *Il10* | CTTACTGACTGGCATGAGGATCA | GCAGCTCTAGGAGCATGTGG |
| *Arg1* | CTCCAAGCCAAAGTCCTTAGAG | GGAGCTGTCATTAGGGACATCA |
| *Rac* | TAGGCCCAGTCGCCCG | GCCAGGTTTTAATATATTCCCCTCG |
| *Cd206* | TTCAGCTATTGGACGCGAGG | GAATCTGACACCCAGCGGAA |
| *Pip5k1b* | GAACCCACGACATCCCGAC | CAGCAGTTGACGACATCTTTGT |
| *Trem2* | ACAGCACCTCCAGGAATCAAG | AACTTGCTCAGGAGAACGCA |
| *Csk* | ATGGTACCAAGTCACAGATCG | TACCAGTTGGGGTCCTTGGT |
| *Csf1r* | TGTCATCGAGCCTAGTGGC | GGTCCAAGGTCCAGTAGGG |
| *Cd36* | TTCTTCACAGCTGCCTTCTGA | CGTGGCCCGGTTCTACTAATTCA |
| *Cd44* | CTTGGCCACCAGAGATCGAG | CGCACTTGAGTGTCCAGCTA |
| *Cd16* | AATGCACACTCTGGAAGCCAA | CACTCTGCCTGTCTGCAAAAG |
| *Pecam1* | AGCCAACAGCCATTACGGTTA | TCGACCTTCCGGATCTCACT |
| *IFNg* | CGAAGCAGATGAATCCGCTGA | TGCGTGGAAATTGGGTGTCC |
| *Mertk* | TTGGGACGTTGGTGGATACG | CTTCTCGGCAGTGCCTCC |
| *Stat1* | AGCTCTGCTCCATACCCTGA | ACCACTGTGACATCCTTGAGA |
| *Apoe* | TTGCTGACAGGATGCCTAGC | CTCCATCAGTGCCGTCAGTT |
| *Csf1* | GTGTCAGAACACTGTAGCCAC | TCAAAGGCAATCTGGCATGAAG |
| *Tlr3* | CTGCGCATATCACAGGCTGA | TCTTTTGGTGCGCGATTGTG |
| *Stat6* | CAATGGTCCTGGTCCAAGTGA | CCAGGCTTTCACACCTCTCCTG |
| *Itgav* | GGATGTTTCTCCTGGTGGGA | TGGCATAATCTCGATTGCCTGT |
| *Gapdh* | CCCTTAAGAGGGATGCTGCC | TACGGCCAAATCCGTTCACA |
| *Ccl1* | CCCCAGCTGTGGTATTCAGG | GGAGGACTGAGGGAAACTGC |
| *Ccl2* | CACTCACCTGCTGCTACTCA | GCTTGGTGACAAAAACTACAGC |
| *Cx3cr1* | CCATCTGCTCAGGACCTCAC | CACCAGACCGAACGTGAAGA |
| *Ccl6* | TCAAGCCGGGCATCATCTTT | CTGCCCTCCTTCTCAAGCAA |
| *Cxcl1* | TCGAGACCATTTACTGCAACAG | CATTGCCGGTGGAAATTCCTT |
| *Cxcl2* | CCCAGACAGAAGTCATAGCCAC | TGGTTCTTCCGTTGAGGGAC |
| *Cxcl5* | TGCCCTACGGTGGAAGTCAT | AGCTTTCTTTTTGTCACTGCCC |
| *Cxcl7* | GCTGATGTGGAAGTGATAGCC | CGATTCTCTTGACGCCAGGG |
| *Cxcl9* | GGAGTTCGAGGAACCCTAGTG | GGGATTTGTAGTGGATCGTGC |
| *Cxcl10* | CCAAGTGCTGCCGTCATTTTC | GGCTCGCAGGGATGATTTCAA |
| *Cxcl11* | TGTAATTTACCCGAGTAACGGC | CACCTTTGTCGTTTATGAGCCTT |
| *Il1a* | TCTCAGATTCACAACTGTTCGTG | AGAAAATGAGGTCGGTCTCACTA |
| *Il1b* | GAAATGCCACCTTTTGACAGTG | TGGATGCTCTCATCAGGACAG |
| *Il1ra* | GTGTCCTGTTTAGCTCACCCA | AGGCACCATGTCTATCTTTTCTTC |
| *Il6* | CTGCAAGAGACTTCCATCCAG | AGTGGTATAGACAGGTCTGTTGG |
| *Il12* | CAATCACGCTACCTCCTCTTTT | CAGCAGTGCAGGAATAATGTTTC |
| *Il18* | AGCAGTCCCAACTAAGCAGTA | CAGCCAGTAGAGGATGCTGA |
| *Spp1* | GAGGAAACCAGCCAAGGACT | AAGCTTCTCCTCTGAGCTGC |
| *Tgfb* | GAGCCAGAACGAGAAGTACCG | CCTCAAGACGAGCAATTTCATCA |
| *Tgfbr1* | TTCCTGAGGAGAAGCTGCG | AACACTGTAATGCCTTCGCC |
| *Il4* | GGTCTCAACCCCCAGCTAGT | GCCGATGATCTCTCTCAAGTGAT |
| *Mfge8* | AACCTAGCCTCCCGTTGTTC | AGACGAGGCGGAAATCTGTG |
| *Crp* | TCGGACTTTTGGTCATGAAGACAT | AGAGAAGACACTGAAGCTGCG |
| *Il1r1* | CACGTGGGTCGTCTGC | GAGAAATCACTGTAGATACCCAGA |
| *Cd14* | ACTGAAGCCTTTCTCGGAGC | TGAAAGCGCTGGACCAATCT |
| *Fcer1g* | TAGCCAGCCGTGAGAAAGCA | GCCAACCTTCAAAGCACAGAG |
| *Fcgr2b* | AAGCAGGTTCCAGACAATCCT | TGGCTTGCTTTTCCCAATGC |
| *Colec12* | CTCAGTCACCATGAAAGACGA | CACTGTGTCCCCTCCTGAAT |
| *Clec7a* | TGGGTGCCCTAGGAGGTTTT | CGGTGAGACGATGTTTGGCT |
| *Tim4* | GCATGGATGGAGAAGGGACTT | TTCATTCTAGGGCACCCTCC |
| **Homo sapiens** | | |
| *ACTB* | ACAGAGCCTCGCCTTTGCC | GATATCATCATCCATGGTGAGCTGG |
| *TREM2* | CTCCGGCTGCTCATCTTACT | AGTCATAGGGGCAAGACACC |
| *CD14* | GAAGACTTATCGACCATGGAGC | AGACGCAGCGGAAATCTTCA |
| *ITGB2* | CAGGGCAGACTGGTAGCAAA | CACTCCTGAGAGAGGACGCA |
| *ITGAV* | CGCTTCTTCTCTCGGGACTC | AGAAACATCCGGGAAGACGC |
| *TIM4* | ATCCCACGTGTCAATGTGGA | AACTGCTGTATCAGATGCTCC |
| *CD36* | TCCTGCAGAATACCATTTGATCCT | TGGTTTCTACAAGCTCTGGTTCTTA |

**Supplementary Figures**


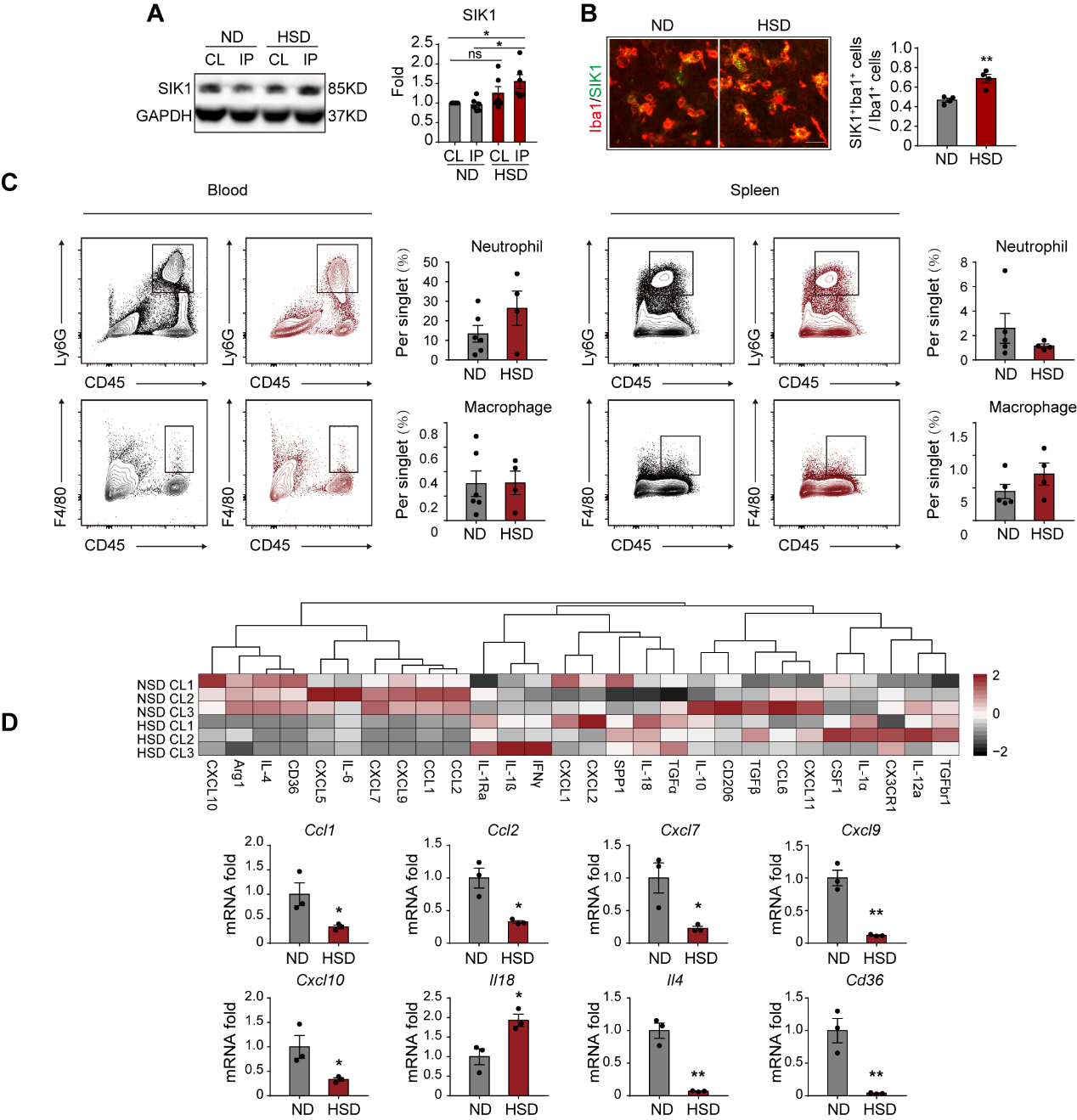


**Supplementary Figure 1. Comparison of inflammatory mediator expression in peripheral blood and contralateral brains of ND and HSD mice.** (**A**) Protein expression of SIK1 in contralateral and ipsilateral hemispheres of stroke mice was assessed with western blot. *N* = 6 mice per group. **P* < 0.05; ns, no significance; *one-way ANOVA*. (**B**) Brain sections were collected from ND and HSD mice at 3d after 60min tMCAO. Expression of SIK1 (green) in Iba1^+^ cells (red) in ischemic lesions was analyzed with immunostaining *in vivo*. *N* = 4 mice per group. ***P* < 0.01 versus ND group in *t-test*. Scale bar, 25μm. (**C**) Macrophages and neutrophils in peripheral blood and spleen were assessed with flow cytometric analysis at 3d after tMCAO. *N* = 4-6 in ND and HSD groups. (**D**) The mRNA expression of inflammatory mediators in the contralateral brains of ND and HSD mice was assessed with RT-PCR. mRNA expression was normalized to the level of the contralateral brain from ND mice. *N* = 3 in ND Cl and HSD Cl groups. **P* < 0.05, ***P* < 0.01, versus ND group in *t-test*. Cl, contralateral.


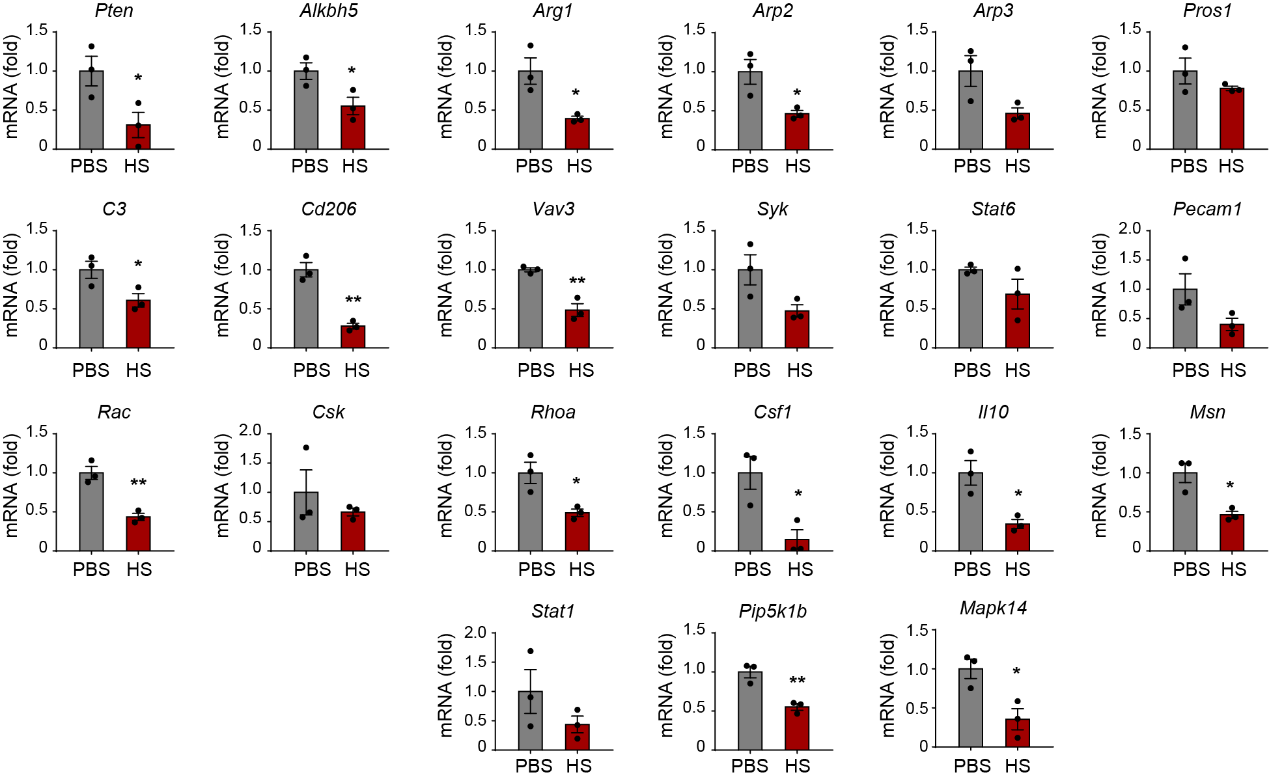


**Supplementary Figure 2. Excess salt downregulates efferocytic molecules in macrophages.** Macrophages cultured in the high salt environment (with 40mM NaCl addition) were subjected to a PCR array of efferocytosis associated molecules. Data are displayed as fold change to macrophages from the PBS-treated group. Data were collected from 3 independent experiments. **P* < 0.05, ***P* < 0.01 versus PBS group in *t-test*.


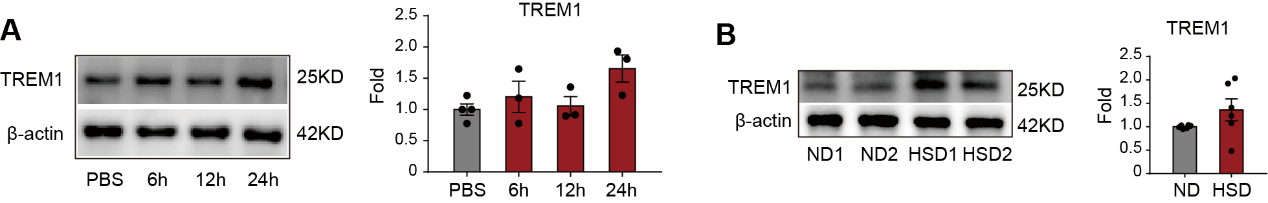
**Supplementary Figure 3. Impact of high salt on TREM1 expression in primary culture macrophage and ischemic brain.** (**A**) Protein expression of TREM1 in macrophage after HS treatment was assessed with western blot. Experiments were repeated three times. No significant difference between the two groups was observed. (**B**) Protein expression of TREM1 in ipsilateral hemispheres of stroke mice was assessed with western blot. *N* = 6 mice per group. No significant difference between the two groups was observed.


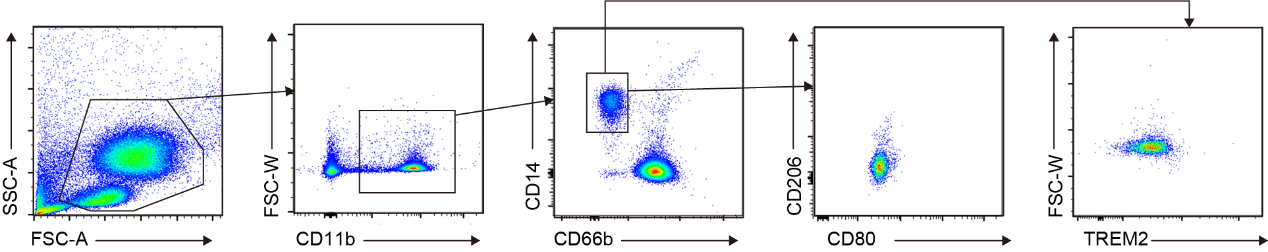
**Supplementary Figure 4. Gating strategy in flow cytometric analysis of TREM2, CD80, and CD206 expression in monocytes of AIS patients.**


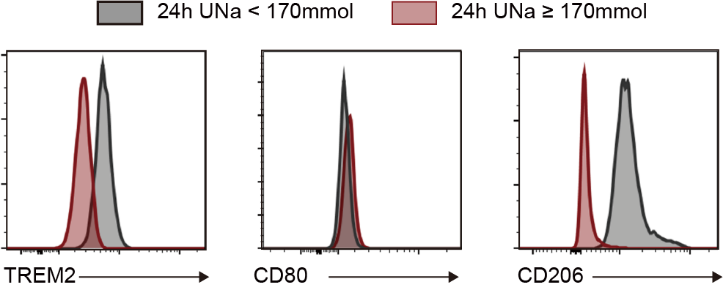


**Supplementary Figure 5. Representative images of TREM2, CD206, and CD80 expression in peripheral monocyte of AIS patients with normal or high urine sodium concentration.**


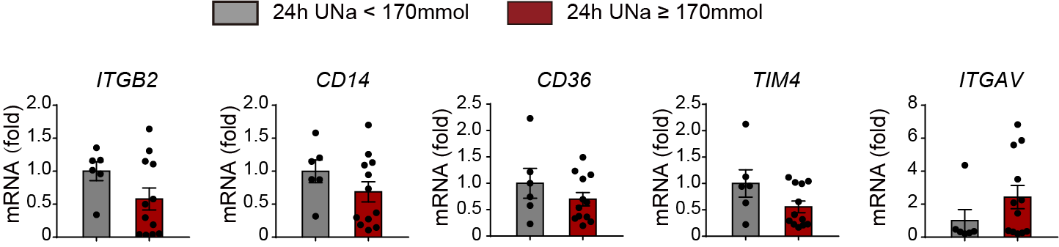


**Supplementary Figure 6.** **PRRs mRNA expression remained no different other than *TREM2* in AIS patients with high salt intake.** Comparison of PRRs mRNA expression in PBMC of AIS patients with normal (*N* = 6) or high urine sodium concentration (*N* = 12). PBMC, peripheral blood mononuclear cells.


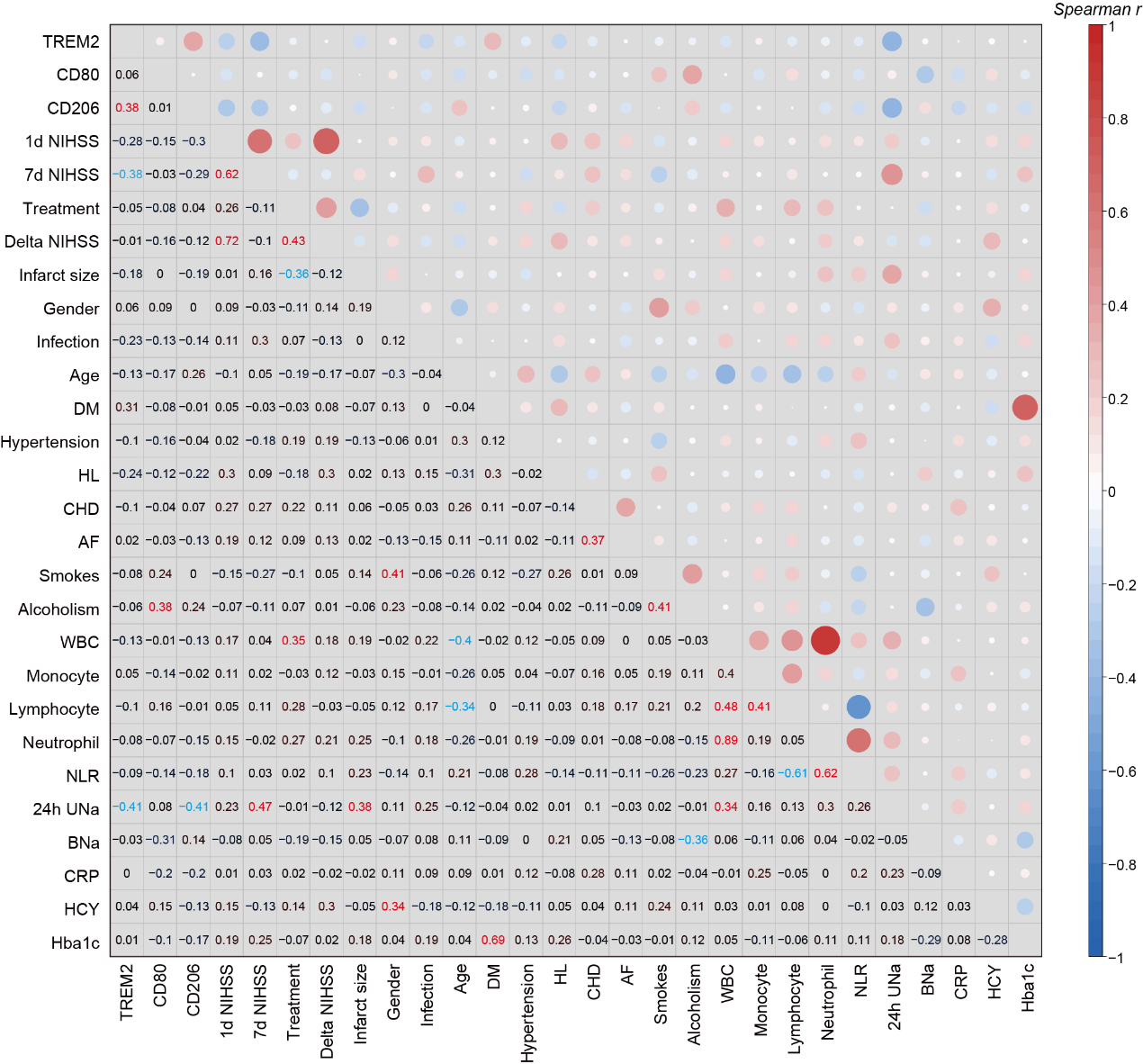


**Supplementary Figure 7. Spearman correlation analysis of TREM2 expression, monocyte phenotypic marker, AIS outcomes and clinic parameters.** *N = 13* in ND stroke patients, *N* = 25 in HSD stroke patients. Red numbers indicate positive correlations, and blue numbers indicate negative correlations. DWI, diffusion-weighted imaging; DM, diabetes mellitus; HL, hyperlipidemia; CHD, coronary heart disease; AF, atrial fibrillation; WBC, white blood cell; NLR, neutrophil-to-lymphocyte ratio; BNa, blood sodium; CRP, C-Reactive Protein; HCY, homocysteine; Hba1c, glycated hemoglobin.


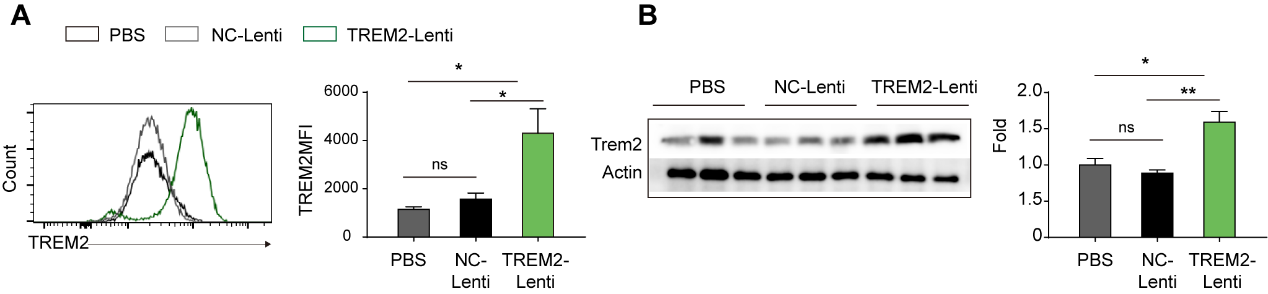


**Supplementary Figure 8. Validation of TREM2 overexpression efficacy in primary cultured macrophages.** Bone marrow-derived primary cultured macrophages were infected with lentiviral vectors carrying TREM2-GFP cDNA (TREM2-Lenti) or control lentivirus carrying GFP only (NC-Lenti). Macrophages were subjected to analysis at 2d after infection. (**A**-**B**) Protein expression of TREM2 was analyzed with flow cytometry (**A**) and western blot (**B**). Experiments were repeated three times. **P* < 0.05, ***P* < 0.01 versus PBS-treated group in *t-test*.


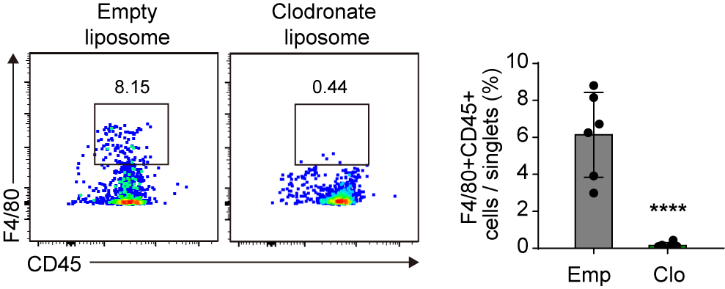


**Supplementary Figure 9. Circulating macrophages and monocytes were depleted by clodronate liposome treatment.** For macrophage depletion, intraperitoneal administration of clodronate liposomes (Liposoma, 10ml/kg) was performed at 3d before tMCAO. Empty liposomes were used as the control (Liposoma, 10ml/kg). Depletion efficacy of circulating macrophages/monocytes in the peripheral blood was confirmed by flow cytometry with F4/80 and CD45 staining. *N* = 6 mice per group. *****P* < 0.0001 versus empty liposome group in *t-test*. Emp, empty liposome; Clo, clodronate liposome.
